# Three-Dimensional In Vitro Hydro- and Cryogel-Based Cell-Culture Models for the Study of Breast-Cancer Metastasis to Bone

**DOI:** 10.3390/cancers10090292

**Published:** 2018-08-27

**Authors:** Laura J. Bray, Constanze Secker, Berline Murekatete, Jana Sievers, Marcus Binner, Petra B. Welzel, Carsten Werner

**Affiliations:** 1 Institute of Health and Biomedical Innovation, Queensland University of Technology (QUT), 60 Musk Avenue, Kelvin Grove 4059, Australia; b.murekatete@qut.edu.au; 2 Centre in Regenerative Medicine, Queensland University of Technology (QUT), 60 Musk Avenue, Kelvin Grove 4059, Australia; 3 School of Chemistry, Physics and Mechanical Engineering, Science and Engineering Faculty, Queensland University of Technology (QUT), 2 George Street, Brisbane 4001, Australia; 4 Translational Research Institute, Mater Research Institute-University of Queensland, 37 Kent Street, Woolloongabba 4102, Australia; 5 Leibniz Institute of Polymer Research Dresden, Max Bergmann Center of Biomaterials, Hohe Straβe 6, 01069 Dresden, Germany; secker@ipfdd.de (C.S.); sievers@ipfdd.de (J.S.); binner@ipfdd.de (M.B.); welzel@ipfdd.de (P.B.W.); werner@ipfdd.de (C.W.); 6 Center for Regenerative Therapies Dresden, Technische Universität Dresden, Fetscherstraβe 105, 01307 Dresden, Germany

**Keywords:** breast cancer, hydrogel, bone metastasis, osteoblasts, 3D model

## Abstract

Bone is the most common site for breast-cancer invasion and metastasis, and it causes severe morbidity and mortality. A greater understanding of the mechanisms leading to bone-specific metastasis could improve therapeutic strategies and thus improve patient survival. While three-dimensional in vitro culture models provide valuable tools to investigate distinct heterocellular and environmental interactions, sophisticated organ-specific metastasis models are lacking. Previous models used to investigate breast-to-bone metastasis have relied on 2.5D or singular-scaffold methods, constraining the in situ mimicry of in vitro models. Glycosaminoglycan-based gels have demonstrated outstanding potential for tumor-engineering applications. Here, we developed advanced biphasic in vitro microenvironments that mimic breast-tumor tissue (MCF-7 and MDA-MB-231 in a hydrogel) spatially separated with a mineralized bone construct (human primary osteoblasts in a cryogel). These models allow distinct advantages over former models due to the ability to observe and manipulate cellular migration towards a bone construct. The gels allow for the binding of adhesion-mediating peptides and controlled release of signaling molecules. Moreover, mechanical and architectural properties can be tuned to manipulate cell function. These results demonstrate the utility of these biomimetic microenvironment models to investigate heterotypic cell–cell and cell–matrix communications in cancer migration to bone.

## 1. Introduction

The development of breast cancer is a multistep process involving epigenetic and genetic cellular changes, irregular interactions within the microenvironment, as well as the deregulation of proliferation, survival, differentiation, and migration [1]. Bone is the most common site for breast-cancer invasion and metastasis, and is often a devastating diagnosis for the patient. The mineralized bone matrix contains numerous growth factors, calcium ions, cell-adhesion molecules, cytokines, and chemokines, which are released during physiological bone remodeling. This makes the bone an attractive site for breast-cancer metastasis. Skeletal metastases disturb the homeostasis of the bone microenvironment and osteolytic or osteoblastic lesions occur [2]. Direct and indirect bidirectional interactions play a major role not only in attracting the breast-cancer cells to bone sites but also in conditioning the cells to adapt to the bone microenvironment. In this manner, numerous chemoattractants, such as transforming growth factor β (TGF-β), vascular endothelial growth factor (VEGF), RANK-ligand, and C-X-C chemokine receptor type 4 (CXCR4) have been associated with the homing of circulating tumor cells to bone and providing a fertile ‘soil’ for these cells in secondary sites [2,3,4]. Moreover, cancer growth and metastatic spread are dependent on the presence of vascular networks. While the general pathophysiology of bone metastasis is known, the specific cellular and molecular mechanisms, as well as physical parameters, that initiate homing of breast-cancer cells to bone sites is still not fully understood. Hence, it is crucial to design relevant cell-culture systems that mimic key features of the in vivo situation in order to unravel the complex interactions between breast-cancer cells and the bone/bone environment.

Tissue engineering has led to the development of three-dimensional (3D) cell-culture scaffolds that better reflect the natural conditions in vivo, when compared with two-dimensional (2D) culture models. It is known that cells change their metabolism and gene-expression patterns, as well as the production of extracellular matrix (ECM), when they are cultured in 2D when compared with 3D [5]. In breast cancer, the 3D microenvironment plays a pivotal role during tumorigenesis and metastasis since the cancer cells interact with each other, with nonmalignant cells, and with the surrounding ECM [6]. Deregulated and dense collagen type I deposits are a major component of tumor ECM and have been found to encourage tumor progression and metastasis [7,8,9]. There have been previous biomaterial-assisted attempts to model breast cancer to bone metastasis, including the use of porous chitosan hydrogel scaffolds with nanocrystalline hydroxyapatite and osteogenic-differentiated mesenchymal stromal cells (MSCs) to mimic the bone microenvironment [10]. In this microenvironment, MDA-MB-231 cells migrated deeply and quickly into an internal scaffold, whereas MCF-7 cells predominantly remained on the surface [10]. Others have developed a microfluidic in vitro triculture system using collagen I gels, which contained embedded primary human osteodifferentiated MSCs [11]. A human umbilical-vein endothelial-cell (HUVEC) monolayer was introduced into the media channels throughout the gel. Afterwards, MDA-MB-231 cells, as a single-cell suspension, were injected into the media channels and extravasation was analyzed. The breast-cancer cells transmigrated through the HUVEC monolayer and into the collagen gel, proliferated, and formed micrometastases in the new microenvironment [11]. In spite of these advances, there is room for improvement in regard to designing in vitro breast-cancer bone-metastasis models that recapitulate the spatial separation of the cells (in order to study cell migration) and the structure of bone, and that are flexible in design with respect to physical and biomolecular properties. Moreover, existing models are still insufficient with respect to the simultaneous application of various cell types to mimic the complex microenvironment [12] and the use of human bone cells, such as osteoblasts, has been lacking.

Here, we developed adaptable hydrogel-assisted biphasic constructs, distinct from previous models, which allow the creation of a physiologically relevant scenario with cocultures of up to four cell types in order to mimic breast-tumor tissue spatially separated from bone. A biohybrid in situ-forming hydrogel, based on the glycosaminoglycan heparin and synthetic four-arm star poly (ethylene glycol) (starPEG) was used for embedding breast-cancer cells. We have recently shown that these hydrogels are able to mimic the breast-tumor microenvironment [13], and that the addition of a collagen I-derived peptide, -GFOGER, promoted a more invasive phenotype in the prostate-cancer cell line, PC3 [14]. The starPEG was also conjugated with matrix metalloproteinase (MMP)-cleavable peptide linkers, allowing for cell remodeling. Moreover, the high affinity of heparin to various biomolecules offers many options for secondary biofunctionalization (e.g., growth-factor loading) [15]. The mechanical properties can be tuned independently of the heparin content of the hydrogel by varying the molar ratio of starPEG to heparin. For primary human osteoblasts (hOBs), culture as a 2D monolayer and within 3D in situ hydrogels were tested. In addition, macroporous starPEG–heparin hydrogels prepared by cryogelation (starPEG–heparin cryogels) in our group [16] provide alternative, easy-to-handle, and robust 3D scaffolds with ideal structural properties to mimic the trabecular bone [17]. Other previous biomaterials used to build 3D scaffolds for bone-tissue engineering include hydroxyapatite, partially demineralized bone, and biodegradable porous polymer matrices; all drawing inspiration from the natural 3D structure of trabecular bone [18,19,20,21]. Bone models often integrate inorganic particles or fibers in hydrogels or other polymer matrix materials (reinforcement); one model using cryogels, made of collagen and nanohydroxyapatite, which were seeded with osteoblasts afterwards, was published by Rodrigues and colleagues [22]. Compared to other cryogel materials, the glycosaminoglycan-based cryogels used in this study not only allow for the binding of adhesion-mediating peptide sequences, but also for the loading and controlled release of a number of cellular-signaling molecules capable of enhancing their bioactivity. Moreover, mechanical and architectural properties within the constructs can be tuned [16,23] to manipulate osteoblastic-cell function in a controlled manner. High porosity and interconnected pores within the cryogels facilitate cell seeding and proliferation, and the cells from bone tissue can deposit their own minerals and provide the scaffold with natural reinforcement. 

The selected systems were combined to create a 3D–2D and in an enhanced 3D–3D coculture system of breast-cancer cells and hOBs in a mineralized matrix. Viability, morphology, and migration of cells within the microenvironments were studied. Vascular and supporting cell types (HUVECs and MSCs) were further added to the hydrogel phase as a triculture with breast-cancer cells to establish a vascularized tumor microenvironment and demonstrate its potential to explore the effects of angiogenesis on breast-cancer migration. This study provides insights in relation to cell–cell and cell–matrix communication, offering promise for future applications of the presented biphasic hydrogel-assisted in vitro models to investigate the mechanisms between breast-cancer cells and the bone metastatic environment.

## 2. Results

We first describe the development, formation, and properties of the single phases (cancer: hydrogel and bone: cryogel) of our biphasic construct, before continuing with the description of the 3D–2D and 3D–3D models.

### 2.1. Establishing Mono- and Triculture 3D In Vitro Models for Breast Cancer by Utilizing In Situ starPEG–Heparin Hydrogels of Different Stiffness and Composition

Different studies have evaluated the influence of matrix elasticity and composition on cellular function [24]. Thus, the response of MCF-7 and MDA-MB-231 cells to in situ hydrogels with varying stiffness was evaluated for the two types of starPEG–heparin networks. As demonstrated in Supplementary Figure S1a, storage moduli for both in situ hydrogels (PEG–MMP, no-ligand: G’ = 0.5, 1.3 and 3.7 kPa, PEG–MMP–GFOGER: G’ = 0.6 and 1.3 kPa) increased with increasing molar ratios (γ) of starPEG and heparin (0.63, 0.76, 1.0, and 1.25) and thus higher cross-linking degrees. The hydrogels formed from HM6 and PEG–MMP–GFOGER, which includes the collagen I-derived GFOGER sequence, were softer for a given molar ratio. As the GFOGER peptide has no free cysteine for the Michael-type reaction with the heparin maleimide [14], only three arms and not four (as in the case of PEG-MMP) are involved in cross-linking. PEG–MMP–GFOGER hydrogels with γ ≤ 0.76 were too unstable for measurements.

In general, all 3D monocultures showed a slower proliferation profile (Presto Blue Assay) than the 2D control (Supplementary Figure S1b–d). PEG–MMP–GFOGER hydrogels with γ = 1.25 were found to be stable for all further experiments. Therefore, γ = 1 was chosen for the PEG–MMP hydrogels to ensure that both hydrogel types were of comparable stiffness (Supplementary Figure S1a), in order to unravel differences in cell behavior due to the different peptide linkers/sequences. MCF-7 cells formed spherical structures within the hydrogels (Supplementary Figure S2a,b,e,f) of 65.23 µm (±1.16 standard error of the mean (SEM); 7 d) and 84.43 µm (±3.16 SEM; 14 d) in diameter (PEG-MMP, γ = 1) (Supplementary Figure S1e). MDA-MB-231 cells proliferated in PEG–MMP γ = 1 hydrogels, but did not show comparable spheroids to MCF-7 cells (29.19 µm ± 3.09 SEM and 34.71 µm ± 3.75 SEM for 7 d and 14 d respectively) (Supplementary Figure S1e). The morphologies of MDA-MB-231 cells were very heterogeneous in the hydrogel (Supplementary Figure S2c,d,g,h). In PEG–MMP–GFOGER γ = 1.25 hydrogels, MCF-7 cells formed spheroids with a diameter of 67.82 µm ± 3.70 SEM (7 d) and 103.00 µm ± 6.03 SEM (14 d) (Supplementary Figure S1e). MDA-MB-231 cells again stayed as smaller single cells or clumps with sizes of 26.22 µm ± 2.13 SEM (7 d) and 29.74 µm ± 1.80 SEM (14 d), respectively (Supplementary Figure S1e). In tricultures of cancer cells with HUVECs and MSCs, endothelial networks formed in both hydrogel types and for both breast-cancer cell types (data not shown).

### 2.2. Establishing 2D and 3D hOB In Vitro Culture Models for Bone

**Figure 1:**
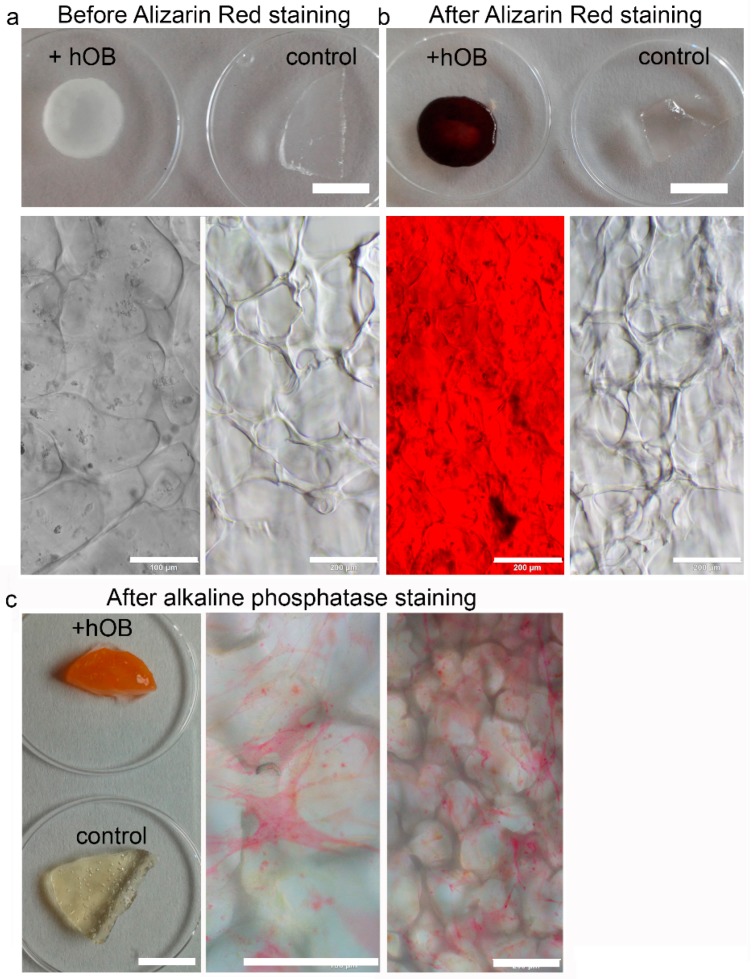
Characterization of the cryogel bone construct. Primary human osteoblasts (hOBs) were cultured for five weeks within the cryogel (including three weeks with mineralization medium). (**a**,**b**) Plate view (upper) and microscopic view (lower) of the staining. (**a**) Microscopic pictures show hOBs within the cryogel before the staining, (**b**) red positive Alizarin Red in cryogels with hOBs and an empty cryogel as a negative control. The empty cryogel completely leached out and no interference with the cryogel material occurred. For alkaline phosphatase (ALP) staining, hOBs were fixed after five weeks of culture in the cryogel. (**c**) Positive ALP staining indicates osteoblast functionality even after five weeks and formation of bone matrix. Furthermore, the macroscopic image demonstrates no interference of the cryogel with the staining solution. (macroscopic scale bar = 5 mm; microscopic scale bar = 200 µm).

**Figure 2:**
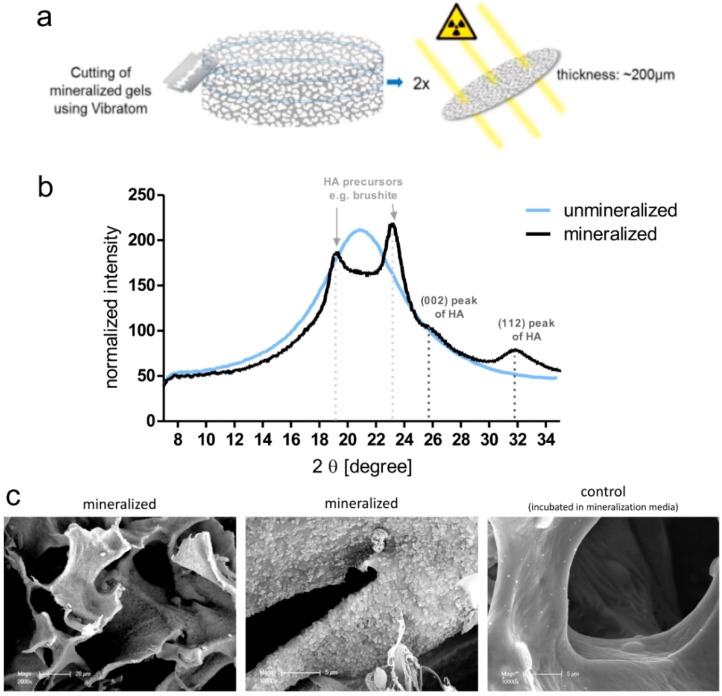
Wide-Angle X-ray Scattering (WAXS) diffraction pattern and scanning electron microscopy (SEM) of mineralized in comparison to nonmineralized cryogels. (**a**) Two different cryogel scaffolds were analyzed. Each cryogel scaffold was cut into thin slices (400 µm swollen state/200 µm dry state) using a vibratome. Afterwards the cut gel discs were dried and then analyzed in their mineral structure with WAXS technique using a NANOSTAR instrument (Bruker, Berlin, Germany). Diffraction patterns of each gel discs were recorded at three different spots within the cryogel discs, one in the middle and two points at the border of the discs, to check if the cryogels were equally mineralized throughout the whole scaffold. (**b**) Diffraction pattern of unmineralized cryogel were found to have one broad peak. This peak probably results from the polymer matrix of the cryogel scaffolds, which when dried show a peak that is characteristic for amorphous polymer materials. Mineralized scaffolds were found to have peaks at four different positions. According to the literature, the principal diffraction peaks of hydroxyapatite (HA) appear at 2 θ values of 25.9° for reflection (002) and at 31.9° for (112) reflection. Additional peaks, e.g., at ~23°, were found, suggesting the presence of other types of calciumphosphate (e.g., brushite, which is often formed as a precursor of HA). Graph shows the average diffraction pattern of two samples. Per sample, diffraction patterns at three different positions were recorded. (**c**) SEM images demonstrate mineralized (left and center) versus nonmineralized (right) cell-free cryogel scaffolds. Scale bars = 20 μm, 5 μm and 5 μm, respectively.

Three different bone models were tested in this manuscript, 2D growth of hOBs on tissue culture plastic, 3D growth in the two different in situ starPEG–heparin hydrogels (no ligand and GFOGER), and 3D growth in macroporous starPEG–heparin cryogels. 2D hOB cultures formed mineralized matrix deposits as evidence by Alizarin Red staining (Supplementary Figure S3). HOBs did not show noticeable proliferation after 7 d and were present as single cells in both PEG–MMP and PEG–MMP–GFOGER hydrogels (Supplementary Figure S1d). When the stiffness of the hydrogels was altered, the hOBs showed no difference in proliferation and only some cell spreading occurred after 14 d (data not shown), making the starPEG–heparin hydrogels appear to be an unfeasible platform for 3D cultures of hOBs. Mineralization was not attempted in the hydrogel cultures. For the third in vitro bone model, starPEG–heparin cryogels were examined to ascertain if they were the appropriate scaffolds for the long-term cultivation and mineralization of hOBs. Images of the dry scaffolds as well as pore structure and pore-size distribution of the phosphate-buffered saline (PBS)-swollen scaffolds are shown in Supplementary Figure S4. First, hOBs were seeded into the cryogel scaffolds in either RGD-functionalized or nonfunctionalized cryogels. Confocal microscopy images (immunofluorescence staining) after 14 d of culture show that the hOBs exhibited their typical elongated phenotype and were distributed throughout the cryogel (Supplementary Figure S5). Most cells appeared to be actively bound to the struts of the cryogel forming a meshlike network. The higher cell density on top and at the bottom of the RGD-functionalized scaffold in comparison to the nonfunctionalized cryogel suggests that the RGD may have helped the cells to bind to the scaffold in their initial adhesion phase (4 h before medium application). Therefore, RGD-functionalized cryogels were used for further experiments. Next, it was investigated if the hOBs had remained functional and established a bonelike environment within the cryogel during culture periods of five weeks (two weeks proliferation, three weeks mineralization). The cryogels were found to have deposition of calcium (Figure 1b) and hydroxyapatite (Figure 2) as a marker for functional osteoblasts, when compared with an empty cryogel control (Figure 1a,b). Bright-red staining also indicated alkaline phosphatase (ALP) activity after five weeks of culture (Figure 1c). 

### 2.3. The 3D–2D In Vitro Model Provides a Method for the Analysis of Paracrine Effects between Breast-Cancer Cells and hOBs

**Figure 3:**
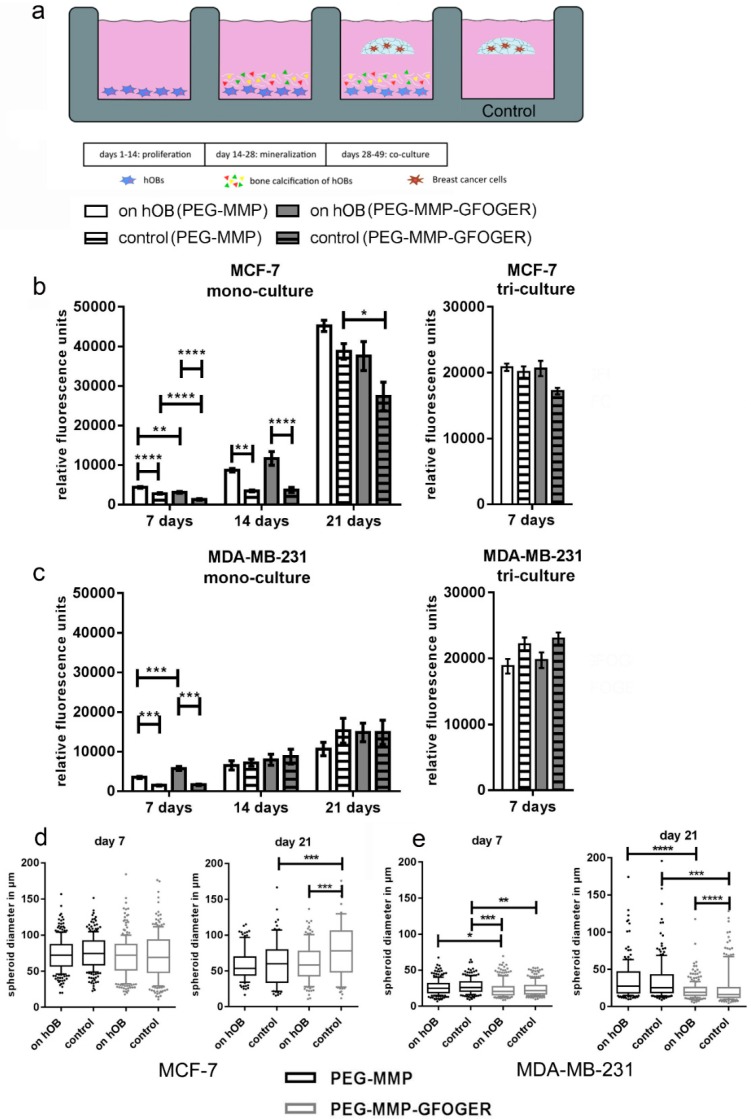
Metabolic activity and tumor spheroid diameter of breast-cancer cells cultured in a 3D–2D coculture model with hOBs. (**a**) Schematic illustration of the indirect 3D–2D coculture setup. The hOBs were stimulated with mineralization medium to deposit calcium and ECM for 14 d. After mineralization, hydrogels with breast-cancer cells or tricultures were cultured as floating constructs in the wells for either 21 d (monocultures) or 7 d (tricultures). (**b**,**c**) Breast-cancer monocultures and tricultures with HUVECs and MSCs were either cultured in PEG–MMP or PEG–MMP–GFOGER hydrogels. HOBs were pregrown in 2D for 4 weeks and hydrogels were cultured on top as floating droplets for an additional 3 weeks. Control groups were cultured without hOBs. Microscopic analyses and PrestoBlue assays were performed after 7 d, 14 d, and 21 d. Bar graphs show mean ± SEM. (**d**) Box plot data represent median values, percentiles (10%–90%) and outliers of spheroid diameters. Monoculture experiments were performed with 6 technical replicates and triculture experiments with 3 technical replicates per condition (n = 3). Asterisks (*) denote statistical significance: * (*p* < 0.05), ** (*p* < 0.01), *** (*p* < 0.001) or **** (*p* < 0.0001) from the control samples.

**Figure 4:**
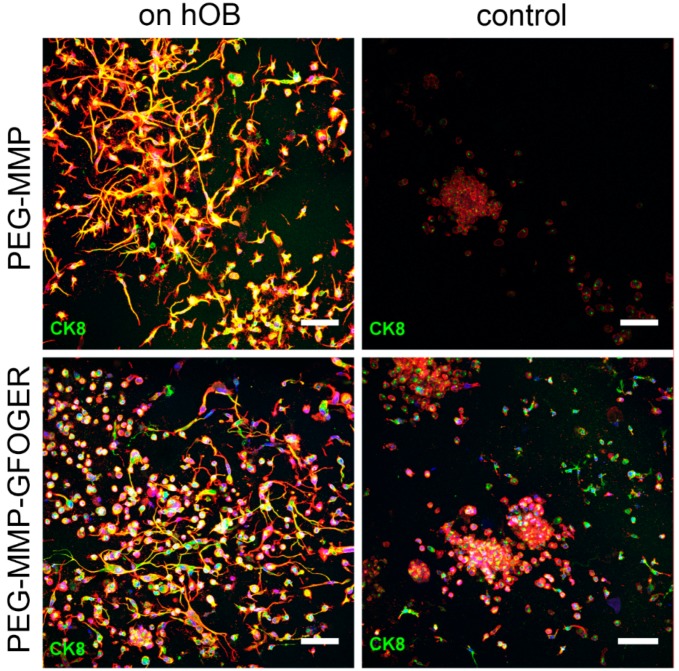
Maximum projection and 3D reconstruction images of MDA-MB-231 cultured on hOBs in a 2D–3D coculture model. Cells were either cultured in PEG–MMP (γ = 1) (**top row**) or PEG–MMP–GFOGER (γ = 1.25) (**bottom row**) hydrogels for 21 d. Cells cultured on hOBs in both hydrogel types showed long protrusions in comparison to more spherical control cells. Staining represents f-actin (red), nuclei (blue), and CK8/18 staining (green). Scale bar = 100 µm.

In the 3D–2D coculture experiments, breast-cancer monocultures and tricultures were grown in in situ hydrogels while exposed to 2D-mineralized layers of hOBs and morphological changes and proliferation were analyzed (Figure 3a). At 7 d, significantly more viable cells were present in hydrogels that were cultured on hOBs; however, this effect diminished over time. No significant differences were found in MCF-7 or MDA-MB-231 tricultures with or without hOBs (Figure 3b,c). The size of tumor spheroids and single cells was measured after 7 and 21 d (Figure 3d,e). While MCF-7 cells demonstrated an increase in metabolic activity over the 21 d experiment (Figure 3b), a slight reduction in spheroid size was observed (Figure 3d). The MCF-7 group maintained single cells and small cell accumulations or spheroids throughout the experiment, rather than increasing spheroid size over time. Although MDA-MB-231 morphologies in the gels were very heterogeneous, significant effects on their spheroid-forming capability could be evaluated. After 7 d, no significant differences were found within the distinct hydrogel type groups, however MDA-MB-231 cells formed larger spheroids in the PEG–MMP hydrogels than in the PEG–MMP–GFOGER hydrogels (Figure 3e). After 7 d, predominantly single cells and small spheroids of a few cells were found (Supplementary Figure S6). At 21 d, MCF-7 cells in the PEG–MMP–GFOGER control group showed significantly larger spheroids than their PEG–MMP counterparts (Figure 3d), while cells cultured within PEG–MMP–GFOGER gels on hOBs showed significantly smaller spheroid size (Figure 3e). These measurements are consistent with the microscopic observations that MDA-MB-231 cells exhibited long cellular protrusions in the coculture and fewer spheroids (Figure 4; Supplementary Figure S6). For both cell types at 21 d, the presence of hOBs significantly decreased spheroid size when compared to the control without hOBs in the PEG–MMP–GFOGER hydrogels (Figure 3d,e). Overall, PEG–MMP–GFOGER monoculture hydrogels were not as stable as their PEG–MMP counterparts during culture periods of 21 d. 

Breast-cancer cell tricultures (MCF-7 or MDA-MB-231 cells either with HUVECs and MSCs embedded within starPEG–heparin hydrogels) were also used in the 2D–3D model and performed for 7 d. Likewise, the network formation was not qualitatively influenced by the indirect coculture with hOBs (Supplementary Figure S7a,b,d,e). No significant differences in proliferation were found for MCF-7 or MDA-MB-231 tricultures in either hydrogel, or in the presence or absence of hOB (Figure 3b,c). Little interaction was observed between the endothelial cells and the MCF-7 cells as revealed by CD31 and CK8 staining after 7 d (Supplementary Figure S7c). MDA-MB-231 cells exhibited spindle-shaped morphology both in control gels and with hOB (Supplementary Figure S7d,e,f).

### 2.4. Analysis of the Influence of Transforming Growth Factor Beta 1 (TGF-β1) and Stromal Cell-Derived Factor 1 (SDF-1) on 3D In Vitro Breast-Cancer Monocultures

**Figure 5:**
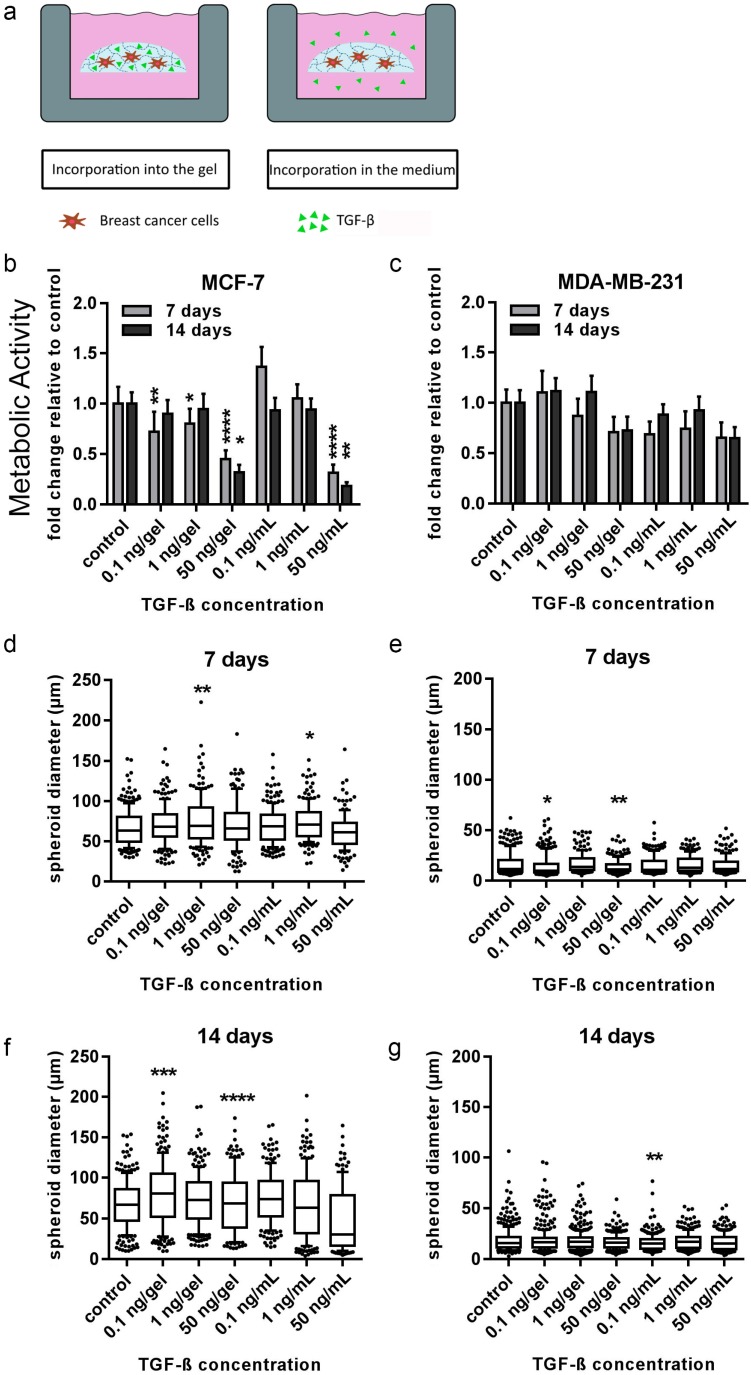
Cell viability and average spheroid diameter of MCF-7 and MDA-MB-231 cells when exposed to transforming growth factor beta 1 (TGF-β1). (**a**) TGF-β1 was incorporated into either the gel or in the media for 14 d of culture. PrestoBlue assays and microscopic analyses were performed (measured by ImageJ). (**b**,**c**) Viability data is presented as fold change relative to untreated control (± SEM). (**d**–**g**) Box plot data represent median values, percentiles (10%–90%), and outliers of spheroid diameters of MCF-7 cells at 7 d (**d**) and 14 d (**f**) and MDA-MB-231 cells at 7 d (**e**) and 14 d (**g**). Experiments were performed three times in triplicate (n = 3). Asterisks (*) denote statistical significance: * (*p* < 0.05), ** (*p* < 0.01), *** (*p* < 0.001) or **** (*p* < 0.0001) from the control samples.

**Figure 6:**
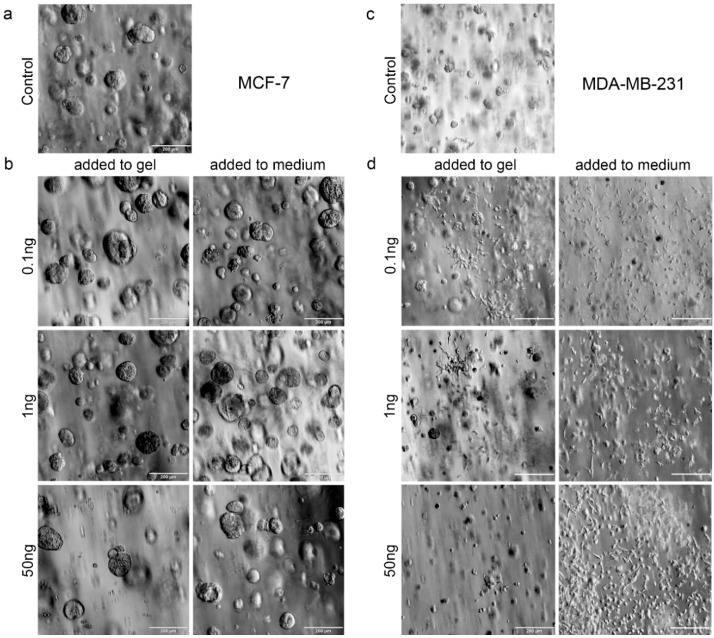
Microscopic pictures of MCF-7 and MDA-MB-231 cells exposed to different concentrations of TGF-β1. Brightfield microscopy images of BC cells after 14 d of exposure to 0 (**a**,**c**), 0.1, 1, or 50 ng (**b**,**d**) of TGF-β either within the hydrogel or added to the cell-culture medium. Scale bar = 200 µm.

We further attempted to mimic the effects of hOBs on breast cancer described in the 3D–2D model in order to dissect the mechanisms involved within the model. TGF-β1, as well as SDF-1, were tested to determine distinct actions of single factors which are known to be important in the bone metastatic microenvironment. Due to the high negative charge of the heparin affecting the diffusion of these factors, they were tested on MDA-MB-231 and MCF-7 cells in three different concentrations either incorporated into the media or into the in situ PEG-MMP hydrogel (γ = 1). Analysis of cell viability revealed that at 14 d, TGF-β1 significantly suppressed MCF-7 growth when added at 50 ng/gel (*p* < 0.05) and 50 ng/mL medium (*p* < 0.01) (Figure 5b). A similar trend was visualized with MDA-MB-231 cells; however, the results were not significant (Figure 5c). Upon TGF-β1 administration, the MDA-MB-231 cells had a heterogeneous population of small spheroids and elongated cells (Figure 5e,g and Figure S9c,d). At 7 d, application of TGF-β1 at 0.1 and 50 ng/hydrogel resulted in a significant decrease in MDA-MB-231 spheroid diameter when compared with untreated samples (Figure 5e). At 14 d, only the spheroid diameter at 0.1 ng/mL TGF-β1 was significantly decreased when compared with untreated controls (Figure 5g). In contrast to the MDA-MB-231 cells, MCF-7 cells formed spheroids (Figure 6a,b). A significant increase in spheroid diameter was found at 7 d for 1 ng/mL or 1 ng/hydrogel when compared with the untreated samples (Figure 5d). After 14 d, spheroids treated with 0.1 ng/hydrogel and 50 ng/hydrogel showed significantly larger diameters when compared with the untreated samples (Figure 5f). 

**Figure 7:**
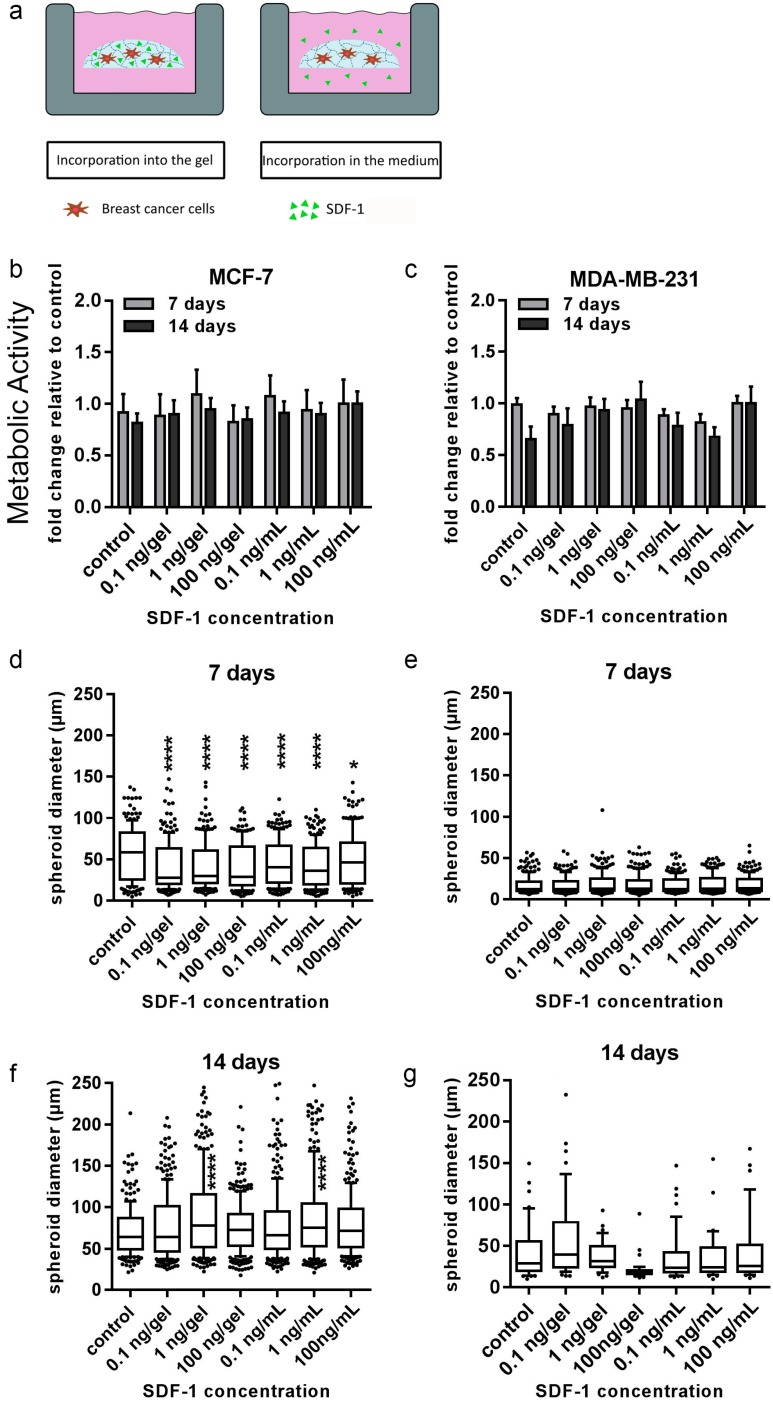
Cell viability and tumor spheroid diameter of MCF-7 and MDA-MB-231 cells when exposed to stromal cell-derived factor 1 (SDF-1). (**a**) SDF-1 was incorporated into either the gel or in the media for 14 d of culture. PrestoBlue assays and microscopic analyses were performed. (**b**,**c**) Viability data are presented as fold change relative to untreated control (±SEM). (**d**–**g**) Box plot data represent median values, percentiles (10%–90%), and outliers of spheroid diameters of MCF-7 cells at 7 d (**d**) and 14 d (**f**) and MDA-MB-231 cells at 7 d (**e**) and 14 d (**g**). Experiments were performed three times in triplicate (n = 3). Asterisks (*) denote statistical significance: * (*p* < 0.05), ** (*p* < 0.01), *** (*p* < 0.001) or **** (*p* < 0.0001) from the control samples.

MCF-7 cells demonstrated significantly smaller spheroid diameters at all concentrations of SDF-1 after 7 d when compared with the untreated control (Figure 7d). After 14 d, the trend was reversed, whereby SDF-1 administration at 1 ng/gel and 1 ng/mL media resulted in significantly larger spheroids than the control samples (Figure 7f). No significant difference in spheroid diameter in MDA-MB-231 cells was found after SDF-1 treatment after 7 d or 14 d; however, the response of MDA-MB-231 cells was quite varied at 14 d (Figure 7e,g). The proliferation of both MCF-7 and MDA-MB-231 cells was not affected by any SDF-1 concentration applied (Figure 7b,c). 

### 2.5. The 3D–3D In Vitro Model Provides a Method for the Study of Cell–Cell and Cell–Matrix Interactions between Breast-Cancer Cells, hOBs, and Their Corresponding Scaffold

**Figure 8:**
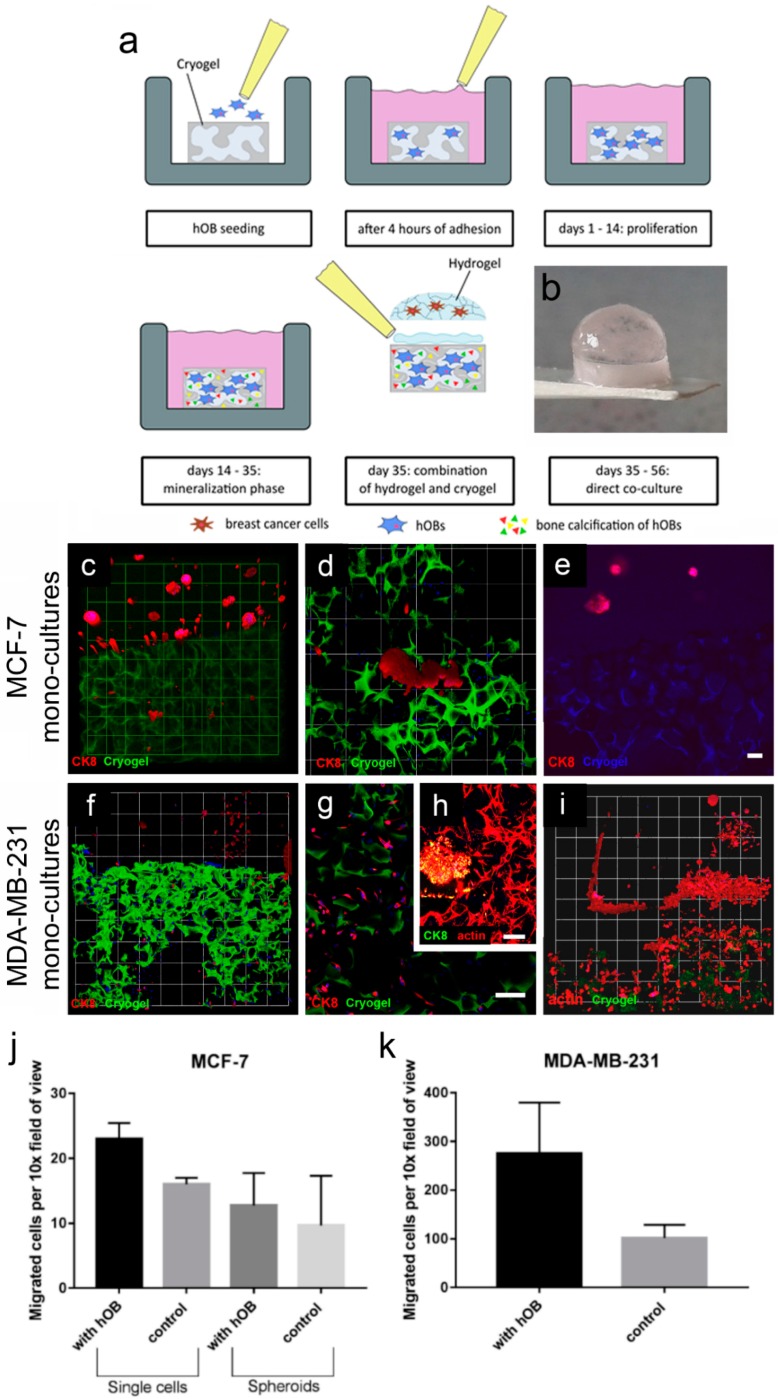
MCF-7 and MDA-MB-231 monocultures grown in a 3D–3D coculture model. (**a**) Schematic showing generation of the 3D–3D coculture model: hOBs were seeded onto cylindrical cryogel scaffolds. After 4 h of initial adhesion, 1 mL of hOB growth medium was added to each sample and they were cultured for 14 d. Next, the hOBs were stimulated in the mineralization phase (days 14–35). (**b**) At 35 d, hydrogels with various cell types were attached on top of the cryogel scaffolds. (**c**–**e**) Representative 3D confocal images show MCF-7 monocultures (CK8, red) grown in a hydrogel–cryogel construction and combined with Alexa Fluor 488-labeled cryogels (green) with (**c**,**d**) hOB or (**e**) an empty cryogel control (autofluorescent, blue). (**c**) A 3D overview of a PEG–MMP hydrogel with tumor spheroids and cryogel with migrated MCF-7 cells (micrometastases) (10×, 1 unit = 155 μm). (**d**) Micrometastases (1 unit = 77.5 μm). (**e**) empty cryogel control (blue) without migrated cells. (**f**) Image shows a 3D overview of a PEG–MMP hydrogel with MDA-MB-231 breast-cancer cells (CK8, red) and cryogel (green) with migrated breast-cancer single cells (10×, 1 unit = 155 µm). (**g**) Migrated elongated cells within the cryogel (CK8, red; cryogel, green). (**h**) HOB meshwork and an MDA-MB-231 micrometastasis within the cryogel (actin, red; CK8, green). (**i**) Migrated MDA-MB-231 cells within the cryogel containing hOBs (CK8, red; cryogel, green). Experiment was performed twice in triplicate (n = 2). Scale bar = 100 µm. (**j**–**k**) Migration of MCF-7 and MDA-MB-231 cells to cryogel in a direct coculture model. The numbers of cells are plotted as number of cells/spheroids per 10× field of view. Experiment was performed once in triplicate (n = 1) and data are presented as mean ± SD.

The final objective of this work was to develop a biphasic 3D–3D scaffold construction to mimic the microenvironment involved in breast-cancer bone metastasis. The designed constructs (Figure 8b) were analyzed in terms of stability with various cocultures and for cell motility and morphology within the 3D–3D coculture constructs (Figure 8a). They possessed an appropriate stability over the culture period of either 21 d (monocultures) or 7 d (tricultures), respectively, as the HUVEC network would not last beyond the 7 d time point. Hydrogels and cryogels remained secured together throughout the culture period (Figure 8b). It was found that the starPEG–heparin hydrogel glue used to attach the hydrogel to the surface of the cryogel was deeply absorbed into the cryogel and filled out part of the pores (Supplementary Figure S5). After 14 d of culture with hOBs, the hydrogel glue was partly degraded within the cryogel and did not deter hOB growth. HOBs formed a fibrous network across the cryogel struts, as visualized by actin staining. 

The confocal images show that both breast-cancer cell lines had migrated after 21 d from the hydrogel (PEG–MMP) towards the cryogels that were seeded with hOBs (Figure 8). MCF-7 cells formed typical spheroids within the hydrogels (Figure 8c). The migrated MCF-7 cells formed a few small tumors in the cryogel scaffold (Figure 8c,d). These micrometastases were layered on the struts of the cryogels instead of forming round spherical tumors. Little migration was found in control experiments with empty cryogels (Figure 8j). The MDA-MB-231 cells which migrated into the cryogel exhibited an elongated morphology (Figure 8h). As expected, MDA-MB-231 cells migrated in a larger number towards the cryogel scaffold in comparison with the MCF-7 cells, and also invaded the empty cryogel controls (Figure 8k). 

All tricultures with both breast-cancer cell lines showed a robust vascularization throughout the gel after 7 d of direct coculture (Supplementary Figure S8a,b,d). Tricultures of MDA-MB-231 cells showed migration as early as after 7 d (Supplementary Figure S8e), but no migration was seen in MCF-7 tricultures after 7 d (data not shown). This was also reflected in the empty cryogel controls (Supplementary Figure S8f). To examine the paracrine effects of hOBs on breast-tumor angiogenesis, 20 spheroids per hydrogel were categorized by aspects of interactions between HUVECs and MCF-7 tumor spheroids. Supplementary Figure S8g–h shows the results of the contact point quantification. HUVECs sometimes formed tumor-invading sprouts or had external contact points, but intravasation events were rarely observed. There was no observable difference of intravasation or tumor contact in both groups. 

## 3. Discussion

It is known that bone is the preferred site of breast-cancer metastasis; however, direct cross-talk between breast-cancer cells and hOBs has not been well explored in vitro. For greater understanding of this complex cross-talk, heterocellular and biomimetic 3D models are required. Here, two biphasic 3D microenvironment models were developed for the study of bidirectional interactions between breast-cancer cells and bone in vitro. Therefore, different from several other approaches, relevant cell types were seeded and cultured in their optimized artificial environment, until breast-cancer tissue and bone-mimicking in vitro systems were formed. Then, these systems were combined as biphasic models. The biomimetic nature of both bone-specific metastasis models was improved by the utilization of primary hOBs instead of modified cell lines or differentiated MSCs in comparison to other models [10,11,25], as it is known that commonly used osteoblastic cell lines differ to an extent from hOBs in terms of proliferation, gene expression, and mineralization [26].

The first model within this study was comprised of breast-cancer cells (MCF-7 or MDA-MB-231) cultured in the enzyme-cleavable starPEG–heparin hydrogels, both as mono- or in coculture with HUVECs and MSCs, indirectly floating above a 2D layer of hOBs. The cell-laden constructs were investigated towards the cellular effect of hOB coculture with breast-cancer cells. The 3D-2D coculture allowed for microscopic control of both components (breast-cancer cells and hOBs) during the whole culture period due to the fact that the bulk hydrogel matrices are optically transparent. For the second model, a 3D–3D model, macroporous starPEG–heparin cryogel templates were utilized to function as bone construct and allowed the coculture of breast-cancer cell lines (embedded within the 3D hydrogels and “glued” to the cryogels), spatially separated but in close proximity to the hOBs. Of the three bone in vitro models tested in this study (2D, hydrogel, and cryogel), the cryogel scaffolds best supported the hOBs. From the ALP and Alizarin Red staining it was found that the hOBs remained functional and established a bonelike environment in the cryogels after mineralization. The cryogels also survived in culture for the complete 5–7 weeks (with or without breast-cancer coculture) and maintained their shape and integrity in culture medium for the entire experiment. The cryogel scaffolds used in the present study are soft (bulk Young’s modulus: 4 ± 3 kPa) but very tough (can be compressed to >90% of their initial volume without losing integrity) [16,23]. Cryogel struts exhibit a rather high local stiffness (Young’s modulus) of approximately 300 kPa as measured by atomic force microscopy (AFM) [23]. The elastic modulus of trabecular bone lamellae is found to be 11.4 ± 5.6 GPa with a comparable measurement method (nanoindentation) [27]; hence, the elastic modulus is much higher in bone compared to the cryogel. Nevertheless, the starPEG–heparin cryogel scaffolds seem to possess attractive features for the 3D in vitro culture of hOBs since they spread and attached to the struts. This is in line with a previous study by Hixon et al., where silk fibroin cryogels were shown to be a promising alternative to traditional bone grafts in bone-tissue engineering [17]. While the starPEG–heparin cryogel model demonstrated effective in vitro mineralized bone formation from human osteoblasts, including the presence of hydroxyapatite and ALP, the model reflects rather early stages of metastasis (when osteolytic activity is limited) [28] and further experiments containing osteoclasts will be needed for its validation. Bone is a calcified tissue that is naturally composed of hydroxyapatite, water, and proteins. These proteins predominantly consist of collagen I, but also noncollagenous proteins, such as fibronectin, osteonectin, osteopontin, and thrombospondin, as well as soluble factors. As yet, the protein composition of our developed construct was not directly compared with native bone. However, soluble factors found in the natural bone matrix such as transforming growth factor (TGF), basic fibroblast growth factors (FGF), and bone-morphogenetic proteins (BMP), are expected to reversibly bind to our cryogel bone model and to be released in a sustained manner as shown for different starPEG–heparin networks in previous studies of our group [29,30,31]. As tumor cells are known to promote osteolytic effects, a bone construct suitable as a more integrative metastasis model would also contain immune cells, osteocytes, and osteoclasts that act to resorb osteoblast-deposited matrix. Future studies will therefore include the detailed analysis and extension of the reported 3D bone model.

In order to draw conclusions about the potential role of bone-cell-secreted factors, the distinct actions of two directly applied single factors (TGF-β1 and SDF-1) on both breast-cancer cell lines embedded in 3D hydrogels were additionally tested. TGF-β1 and SDF-1 (CXCL-12) and its G-protein-coupled transmembrane receptor, CXCR4, have been shown to be implicated in breast cancer and bone metastasis at many levels [32,33]. Moreover, SDF-1 has been shown to be constitutively expressed in organs like lung, liver and bone, where metastases frequently occur. In the current study, TGF-β1 stimulated a transformation of hydrogel-embedded MDA-MB-231 cells into a more invasive and migratory morphology, which is consistent with several reported studies [34,35]. Cui and colleagues [36] showed that TGF-β1 acts early as a tumor suppressor, but later accelerates epithelial to mesenchymal transition (EMT) in a mouse-skin model. Hence, the cells transformed into a more invasive morphology. Moreover, consistent with findings of Manni and colleagues in a soft agar culture, MCF-7 cells in this study formed fewer tumors in comparison to the control groups upon TGF-β1 administration in a dose-dependent manner, most likely caused by the growth-suppressive effect [37]. SDF-1 was expected to trigger an invasive phenotype in hydrogel-embedded breast-cancer cells used in the current study [38,39]. Nevertheless, none of the two investigated breast-cancer cell lines showed significant changes in proliferation or morphology to SDF-1 within the starPEG–heparin hydrogels after 14 d. It is conceivable that the SDF-1 distribution in the current setup may have been too diluted to induce migration, or the affinity of heparin for biomolecules such as SDF-1 rendered the cytokine gradient nonexistent. 

Both biphasic coculture models indicated cross-talk between the 3D-engineered breast-cancer tissuelike and the bonelike environment. MDA-MB-231 cells in the 3D hydrogel environment showed long protrusions suggesting the presence of hOB-secreted factors, which enhance invasiveness, as demonstrated for TGF-β1 in the current work. The morphological changes were more pronounced on hOBs when compared with the experiments where TGF-β1 was directly added to the hydrogel-embedded cancer cells. However, no major differences in morphology were visualized between control and collagen I-derived GFOGER functionalized hydrogels. Other authors also found long protrusions [25] and increased proliferation [40] of breast-cancer cells in coculture with osteoblast-like tissue, or hOB-conditioned medium, respectively; however, molecular analysis could give more insights into the active signaling pathways. For the 3D–3D coculture of hOB-laden cryogels in contact with breast-cancer-cell-laden hydrogel constructs, both breast-cancer cell lines migrated to the 3D-engineered bonelike environment and formed micrometastases. Furthermore, this model illustrated the differences of the inherent migratory behavior of different breast-cancer cell lines. Highly metastatic MDA-MB-231 cells migrated quickly (around 7 d) and in a higher number into the bone construct, whereas low metastatic MCF-7 cells needed more time for mobilization and fewer cells invaded the bonelike cryogel. This concurs with previous reports using Matrigel, where MDA-MB-231 [41], but not MCF-7 [42], cells were to be more invasive. Furthermore, in both cases, migration to the cryogel construct was higher in the hOB-modified microenvironment, probably due to hOB-secreted cytokines. 

Lastly, it is widely recognized that vascular cells are essential components in the tumor microenvironment and play a pivotal role in cancer progression. Previous studies have used bone-marrow MSCs, HUVECs, and breast-cancer cells to mimic the bone microvascular niche in order to investigate breast-cancer dormancy. It was shown that stable microvasculature induced and sustained dormancy, whereas sprouting endothelium promoted tumor growth [31]. Other studies demonstrated that MSCs can greatly enhance the metastatic behavior of MCF-7 and MDA-MB-231 [32]. MDA-MB-231 were observed to fuse with MSCs, forming hybrid-cell populations in another report [33]. These findings illustrate that MSCs can interact with breast-cancer cells and the MSCs could cause the morphological changes of the MDA-MB-231 cells, and thus similar interactions could be investigated with the model presented herein. In this way, we also investigated the interactions of HUVECs with breast-cancer cells in the presence and absence of hOBs; however, no significant differences in migration or endothelial-tumor interactions were found.

## 4. Materials and Methods 

### 4.1. Preparation and Characterization of Hydrogels

Two types of cysteine-terminated starPEG with a different peptide-functionalization pattern were used in this study: a PEG (MMP)_4_ (PEG–MMP; molecular weight (MW): 15,500 Da), and a PEG (MMP)_3_(GFOGER)_1_ (PEG–MMP–GFOGER; MW: 15,950 Da). The former consists of a four-armed starPEG, each arm modified with an apopeptide (GCGGPQGIWGQGCG), which includes an MMP-cleavable site (highlighted as bold text). Synthesis details are given in Reference [43]. Secondly, the PEG–MMP–GFOGER has three arms modified by the MMP-cleavable sequence and one arm modified by a collagen I-derived peptide, one of the main components of tumor ECM (sequence: CWGOPGFOGER, M_w_ = 15,945 Da), as reported recently [14]. Heparin, as the second gel component, was functionalized with 6 maleimide groups (HM6; M_w_: 15,000 Da) as described previously [43]. The mechanical properties of the hydrogel were adjusted by varying the molar ratio (γ) of the starPEG and HM6. 

For the rheological measurements, in situ hydrogels were prepared as described previously [13]. In order to examine the rheological behavior of the gels, the storage modulus (G’) was measured. Gels were swollen overnight in PBS at 37 °C and immediately punched out (0.8 cm) before measurement. Oscillating measurements were carried out on a rotational rheometer ARES LN2 (TA Instruments, Eschborn, Germany) at room temperature. The experimental setup was a plate/plate arrangement (sanded steel plates, diameter 0.8 cm). All gels were individually placed between the stationary plate and the flexible plate and compressed to about 90% of their initial height. The resulting measuring force was noted and was between 0.2 and 8.5 g. The gels were then exposed to shear frequencies of 1–100 rad/s (strain amplitude: 2%). The mean values of the storage moduli were calculated (n = 2, 11 individual measurements of a gel).

### 4.2. Preparation and Characterization of Cryogels

Cryogels were prepared as described previously [16,23]. Network formation via chemical crosslinking (carbodiimide chemistry) of 4-arm amino-terminated starPEG (M_w_: 10,000 g/mol; JenKem Technology, Plano, TX, USA) and heparin (M_w_: 14,000 g/mol; Merck, Germany) was combined with cryogelation technology. In brief, heparin and amino end-functionalized starPEG were each dissolved in one-third of the total volume in ice-cold MilliQ water. For fluorescently-labeled cryogels, 1% (*w*/*w*) Alexa Fluor^®^ 488-labeled heparin (prepared from Alexa Fluor^®^ 488, Thermo Scientific, Karlsruhe, Germany) was added. Similarly, 1-ethyl-3-(3-dimethylaminopropyl) carbodiimide (EDC) and *N*-hydroxysulfosuccinimide (sulfo-NHS) (both Sigma-Aldrich, Steinheim, Germany, molar ratio 2:1) were each dissolved in one-sixth of the total volume. Based on the amount of NH_2_ groups of starPEG, a two-fold molar excess of EDC was used. To activate the heparin carboxyl groups, EDC and sulfo-NHS were added for 15 min to the heparin solution at 4 °C. Afterwards, the starPEG solution and heparin solution were mixed well. This reaction mixture was pipetted into the cavities of a 96-well plate (350 µL per well) and frozen at –20 °C overnight before lyophilization (24 h). For the present study, a molar ratio of starPEG to heparin of γ = 2 and a total precursor concentration of 11.7% (*w*/*w*) were used. The resulting cylinders could be cut in slices of 1 mm thickness and punched into 3 mm diameter discs. Finally, cryogel discs were washed and swollen in PBS before sterilizing them with ProClin (Supelco, Sigma Aldrich, Steinheim, Germany, 0.04% in PBS) overnight. Following 3 washing steps with PBS, part of the discs were activated for 1 h with 50 mM EDC/25 mM sulfo-NHS solution in 67 mM phosphate buffer (pH 5) for functionalization with a fibronectin-derived adhesion-mediating peptide sequence (to test whether this improves cell adhesion). After washing with borate buffer (100 mM, pH = 8, 4 °C), these discs were incubated with 300 µL of a 50 µg/mL solution of H_2_N-GWGGRGDSP-CONH_2_ (M_w_: 887 g/mol; synthesized in house) in borate buffer for 3 h at room temperature. Functionalized cryogels were subsequently washed with PBS and preincubated with DMEM overnight [16].

The global and local mechanical properties of the PBS-swollen cryogel scaffolds were obtained by means of uniaxial-compression tests and atomic force microscopy-based nanoindentation as reported elsewhere [16,23]. Morphological features and pore-size distribution were determined from cross-sectional confocal images of fluorescently labeled cryogels as described in References [16,44].

### 4.3. Cell Culture

The breast-cancer cell lines MCF-7 and MDA-MB-231 were obtained from the Deutsche Sammlung für Mikroorganismen und Zellkulturen (DSMZ; Braunschweig, Germany) and used within 10 passages. MCF-7 cells were cultured in an RPMI medium supplemented with GlutaMax (Life Technologies, Darmstadt, Germany), 10% fetal bovine serum (FBS, Hyclone Thermo Scientific, Schwerte, Germany), 1× MEM nonessential amino acids (Life Technologies), 1 mM sodium pyruvate (Sigma Aldrich, Steinheim, Germany), 1% penicillin/streptomycin (PS), and 0.1% human insulin (both Life Technologies). MDA-MB-231 cells were cultured in a DMEM medium (Life Technologies) supplemented with 10% FBS and 1% PS. Primary hOB were obtained from PromoCell (Heidelberg, Germany) and cultured in an alpha-MEM medium with nucleotides (Life Technologies) supplemented with 10% FBS and 1% PS. They were used within 8 passages. HUVECs were isolated from umbilical veins as previously described [45], and cultured on fibronectin-coated flasks. The endothelial-cell growth medium (ECGM, PromoCell) contained a supplemental mix with 2% FBS (SupplementMix C-39215, Promocell, Heidelberg, Germany). MSCs were derived from healthy volunteer donors after informed consent (Universitätsklinikum Dresden) as described previously [46], and cultured in DMEM supplemented with 10% FBS, 1% PS, and 1% of antibiotic/antimycotic solution 100× (Sigma Aldrich, Steinheim, Germany). HUVECs and MSCs were used within 6 passages. 

### 4.4. Monoculture 3D In Vitro Model for Breast Cancer

Either PEG–MMP or PEG–MMP–GFOGER in combination with HM6 was used for hydrogel formation. Each individual casted hydrogel that had a total volume of 20 µL and all precursors were dissolved in PBS. Monoculture tumor models were prepared as described previously [13]. In order to evaluate the optimal stiffness of the hydrogels for breast-cancer-cell cultivation, gels of a varying stiffness were prepared. The molar ratios of the starPEG to heparin were γ = 0.76, γ = 1, and γ = 1.25. MDA-MB-231 and MCF-7 monocultures were seeded in a density of 1 × 10^4^ cells/gel and furthermore into each well of a 24-well plate as a 2D tissue culture plastic (TCP) control. In brief, 10 µL of the starPEG solution and 10 µL of the HM6-cell suspension were promptly mixed in a 0.5 mL microcentrifuge tube. Before polymerization, the solution was immediately transferred to a microscope slide coated with a hydrophobic silan (Sigmacote®, Sigma Aldrich, Steinheim, Germany). The resulting gel drops polymerized and were carefully transferred after 2 min into 1 mL of appropriate medium in a 24-well plate. Hydrogels were incubated at 37 °C and 5% CO_2_. 50% of the medium was changed twice a week. PrestoBlue Assay and microscopic analysis were performed after 7 and 14 d.

### 4.5. Triculture 3D In Vitro Model for Breast Cancer

Triculture tumor models were prepared as described previously [13,14]. The heparin fraction was functionalized with 2 mol of a fibronectin-derived adhesion-mediating peptide sequence, H_2_N-GCWGGRGDSP-CONH_2_ (RGD-SP; M_w_ 990; synthesized in house) per mole of HM6. Afterwards, vascular endothelial growth factor (VEGF; PeproTech, Hamburg, Germany), stromal cell-derived factor 1 (SDF-1; Miltenyi Biotec, Bergisch Gladbach, Germany), and fibroblast growth factor 2 (FGF-2; Miltenyi Biotec) were added to stimulate HUVEC-tube formation, and mixed at a concentration which corresponds to a final concentration of each 5 µg/mL per casted gel. Breast-cancer cells (1 × 10^4^ cells/gel), MSCs (1.2 × 10^4^ cells/gel), and HUVECs (1.2 × 10^5^ cells/gel) were each added into the heparin–RGD-growth factor mixture. Finally, the HM6-cell suspension was mixed with PEG–MMP or PEG–MMP–GFOGER in a volume ratio of 1:1 and 20 µL gels were casted onto a Sigmacote-treated microscope slide. For angiogenesis triculture experiments, PEG–MMP hydrogels were created with a crosslinking degree of γ = 0.76 and PEG–MMP–GFOGER with γ = 1, which equated to equal stiffness, due to PEG–MMP–GFOGER having one less arm available for crosslinking. Tricultures were cultured in 1.5 mL of ECGM at 37 °C and 5% CO_2_. 

### 4.6. Development of In Vitro Models for Bone

For 2D experiments, hOBs were seeded into 6-well tissue culture plates at a density of 5 × 10^4^ cells per well. Initially the hOBs were cultured for 10–14 d in a 3 mL growth medium until they reached 90%–100% confluency. Then, the entire medium was replaced by mineralization medium (with Supplement mix (C-39616), Promocell, Heidelberg, Germany). Medium exchange (50%) was performed twice a week for 14 d. 

In order to evaluate the potential use of 3D in situ hydrogels for hOB cultivation, gels of a varying stiffness were prepared. The molar ratios of the hydrogels were γ = 0.76, γ = 1, and γ = 1.25. 2 × 10^4^ cells/gel were cultivated for hOB hydrogel monoculture experiments. In brief, 10 µL of either PEG solution and 10 µL of the HM6-cell suspension were promptly mixed in a 0.5 mL microcentrifuge tube. Before polymerization, the solution was immediately transferred to a microscope slide coated with a hydrophobic silan (Sigmacote®, Sigma-Aldrich). The resulting hydrogel drops were carefully transferred after 2 min into 1 mL of appropriate medium in a 24-well plate. Hydrogels were incubated at 37 °C and 5% CO_2_. 50% of the medium was changed twice a week. 

Alternatively, hOBs were cultured on cryogel discs for 3D experiments. The cryogels were preincubated with hOB growth medium prior to cell seeding. Then, the cryogels (~0.64 cm diameter, swollen) were transferred onto a sterile sheet of filter paper for approximately 10 s to remove the medium from the macropores. The semidry cryogel was put into a 96-well plate and 15 µL of hOB suspension with 1 × 10^5^ cells were applied on top of the cryogels (Figure 5a). Afterwards, the cryogels were incubated for 4 h at 37 °C and 5% CO_2_ to allow cell adhesion. The cryogels were transferred into a 24-well plate and each well was filled with 1 mL of hOB growth media. Cryogels were incubated at 37 °C and 5% CO_2_. After 14 d of proliferation the medium was changed to mineralization medium and the cryogels were maintained therein for another 21 d. Twice a week, 50% of medium was exchanged. PrestoBlue Assay and microscopic analysis were performed after 7 and 14 d. For all bone models, mineralization and functionality were assessed by Alizarin Red and Alkaline Phosphatase staining.

### 4.7. PrestoBlue Viability Assay

A 1:10 dilution of the 10× concentrated PrestoBlue dye (Invitrogen, Thermo Scientific, Karlsruhe, Germany) was prepared in serum-free RPMI medium. After the removal of media, 300 µL of the solution was added to the samples (24-well plate). Then, the cells were incubated for 45 min at 37 °C. Afterwards, 100 µL of the solution was transferred at least in two technical replicates to a 96-well plate. Finally, fluorescence was measured by a Tecan GENios plate reader (Tecan Deutschland GmbH, Mainz-Kastel, Germany) at a wavelength of 590 nm.

### 4.8. Immunofluorescence Staining

After culturing, hydrogel and cryogel samples were washed in PBS with calcium and magnesium (Sigma Aldrich, Steinheim, Germany) for 3 min and fixed for 15 min with 500 µL of paraformaldehyde (PFA, 4%, Fluka, Deisenhofen, Germany). Then, the samples were washed in 1% goat serum (Jackson Immuno Research Lab. Inc, Ely, UK) and permeabilized by 0.1% Triton X-100 (Sigma Aldrich, Steinheim, Germany) in PBS. Next, the samples were blocked for 2 h on a shaking device (5% goat serum, 0.1% Triton X-100 in PBS). All washing and antibody-incubation steps were performed with 1% goat serum and 0.02% Triton X-100 in PBS on a shaking device while covered in aluminum foil. A 1:100 primary antibody dilution of Ki67 (Life Technologies), Cytokeratin 8/18 (Dianova, Hamburg, Germany), or CD31 (BD Biosciences, Heidelberg, Germany) in 1% goat serum was prepared and 400 µL was added to each sample. Negative controls were incubated only with 1% goat serum with 0.02% Triton X-100 in PBS. On the next day, hydrogels and cryogels were washed for at least 8 h. The washing buffer was changed 3 times per day. Afterwards, a secondary Alexa Fluor 488 or 633 antibody (Life Technologies) was applied to the samples at a 1:200 dilution in combination with Phalloidin ATTO 633 or 488 (1:100 dilution, 400 µL) overnight. Next, the samples were washed 3 more times over 8 h and stained with Hoechst 33,342 nuclear dye (1 ng/mL in PBS, 30 min). Finally, the samples were stored in PBS at 4 °C covered in foil until they were visualized using a Leica Confocal SP5 microscope (Leica Microsysteme Vertrieb GmbH, Wetzlar, Germany).

### 4.9. Microscopy Image Analysis

Microscopy pictures were taken using either an inverted brightfield Olympus IX73 or a Leica SP5 confocal microscope. Image analysis was performed with Fiji (ImageJ, NIH) or Volocity® 3D Image Analysis Software (v 6.3, Perkin Elmer, Hamburg, Germany). The measurement of spheroid size was conducted using brightfield microscope images by drawing a line across the center of the spheroid. Only cells/spheroids in focus for each image were measured. In 3D–3D coculture experiments, analysis of interactions between tumor spheroids and HUVECs in the hydrogel construct was performed. Therefore, contact points of MCF-7 and HUVECs were classified as (1) internal contact, (2) external contact, and (3) no contact. MCF-7 cells were easily identified based on their uniform spherical shape and HUVECs were stained for CD31. Analysis was performed directly using the Leica SP5 confocal microscope (20 spheroids per hydrogel).

### 4.10. Alizarin Red Staining for Mineralization

An Alizarin Red S staining solution with 2 mg Alizarin Red S salt (Fluka Chemie GmbH, Buchs, Switzerland) dissolved in 100 mL distilled water was used. Since the pH is critical to the reaction, the pH was adjusted to 4.1–4.3 with HCl and NH_4_OH. After fixation as described above, the cells were washed twice with distilled water and the samples were covered with 400–500 µL Alizarin Red S staining solution. The samples were incubated for 45 min covered in foil on a shaking device. Finally, the samples were washed 4 times with 1 mL distilled water and the color change was assessed macroscopically and with the brightfield microscope Olympus IX73 (Olympus Life Science, Hamburg, Germany).

### 4.11. Alkaline Phosphatase Staining

The cell-seeded cryogels were fixed for 2 min with 4% PFA. Afterwards, the scaffolds were washed with PBS and could be stored at 4 °C. Before staining, the scaffold was rinsed with 0.1 M Tris HCl (pH = 9.2, Trizma base minimum (Sigma Aldrich, Steinheim, Germany) in deionized water). Then, the following substances were also dissolved in 10 mL Tris HCl in order to prepare the staining solution: 2 mg of naphtol AS-MX phosphate (Sigma Aldrich, Steinheim, Germany) and 10 mg of Fast Red TR (Sigma Aldrich, Steinheim, Germany). An appropriate volume was transferred into every well and the samples were incubated for 20 min in a shaking device. At the end, the red precipitate was analyzed on a brightfield microscope Olympus IX73 (Olympus Life Science). 

### 4.12. Scanning Electron Microscopy

To analyze the morphology of the mineral in the cryogel scaffolds, SEM samples were fixed in glutaraldehyde and dried via critical point drying with a CPD 030 apparatus (BAL-TEC AG, Balzers, Liechtenstein). Samples were coated with gold using a SCD 050 sputter coater (BAL-TEC, Balzers, Liechtenstein) for 40 s at 40 mA and imaged with a XL30 ESEM-FEG microscope (Philips, Amsterdam, The Netherlands) in high vacuum mode with a secondary electron detector.

### 4.13. Wide-Angle X-Ray Scattering (WAXS)

For WAXS measurements, cryogel samples were fixed in 4% paraformaldehyde and then cut into small discs using a semiautomatic vibrating blade microtome (Vibratom VT1200 from Leica Biosystems, Nussloch, Germany). Afterwards, samples were dried (ethanol dilution series) to obtain cryogels disc with a height of about 200 µm (dry state). WAXS measurements were carried out using the laboratory X-ray diffraction system NanoStar from Bruker AXS (Karlsruhe, Germany) with a wavelength of 0.154 nm. The WAXS patterns were corrected for instrument-related background scattering and the integration of the two-dimensional scattering data gave the intensity (I) as a function of the scattering vector (2 theta). For all samples, WAXS patterns from the middle and boarder of the cryogel discs were collected to analyze the distribution of the mineral throughout the scaffold. 

### 4.14. Analysis of the Influence of the Cytokines TGF-β1 and SDF-1 on 3D Breast-Cancer Monoculture Models

All experiments were carried out with PEG–MMP hydrogels (γ = 1). Monocultures of MCF-7 (1 × 10^4^ cells/gel) and MDA-MB-231 (2 × 10^4^ cells/hydrogel) were prepared as described above. Different concentrations of TGF-β1 (PeproTech, London, UK) or SDF-1 were added either into the hydrogel or into the medium. Both growth-factor delivery methods were utilized in order to determine the effect of the growth factors either in direct contact with the cells (inside the hydrogel) or when a gradient was created (in the medium). For the incorporation of the factor into the gels, TGF-β1 was mixed into the HM6 fraction according to the above-mentioned protocol. Control gels were cultured without any TGF-β1 or SDF-1. Hydrogels were incubated at 37 °C and 5% CO_2_. Medium was changed twice a week (50% of volume) and medium with TGF-β1 or SDF-1 was freshly prepared each time. PrestoBlue Assay and microscopic analysis was performed after 7 and 14 d. 

### 4.15. Statistical Analysis

All statistics were performed using GraphPad Prism 5.0. One-way ANOVA analysis was utilized to investigate if there was a statistically significant difference between groups. Either Tukey’s (comparison of all groups) or Dunnett’s (comparison with one control group) multiple comparison post-tests were also applied. Results were expressed as mean ± standard deviation (SD) or standard error of the mean (SEM). The difference between two groups was considered statistically significant when *p* < 0.05. Asterisks denote statistical significance: * (*p* < 0.05), ** (*p* < 0.01), *** (*p* < 0.001) or **** (*p* < 0.0001).

### 4.16. 2D–3D In Vitro Model for Breast-Cancer Metastasis to Bone

After 14 d of 2D hOB mineralization, in situ breast-cancer starPEG–heparin hydrogels were prepared as described (Supplementary Materials and Methods) and cultured as floating constructs in the 2D wells with hOBs. The γ = 1 PEG–MMP and γ = 1.25 PEG–MMP–GFOGER monoculture hydrogels contained 1 × 10^4^ breast-cancer cells per gel. Furthermore, for triculture experiments, 1.2 × 10^5^ HUVECs and 1.2 × 10^4^ MSCs were added with the breast-cancer cells in γ = 0.63 PEG-MMP or γ = 0.76 PEG-MMP-GFOGER hydrogels. The medium for the hydrogel culture period was adapted to the hydrogel cell type (monoculture: breast-cancer medium; triculture: endothelial-cell growth medium; ECGM). Hydrogels were incubated at 37 °C and 5% CO_2_. Cell viability and breast-cancer spheroid diameter were measured after 7, 14 and 21 d. For the PrestoBlue assay, hydrogels were carefully transferred into a new 24-well plate in order to eliminate the presence of hOBs. For spheroid measurements, the diameter of all spheroids from 3 photographs per hydrogel was measured using ImageJ (NIH). Triculture models were fixed after 7 d, monoculture models after 21 d.

### 4.17. 3D–3D In Vitro Model for Breast-Cancer Metastasis to Bone

Cell-seeded hydrogels and cryogels were combined in order to examine the interactions of breast-cancer cells and hOBs when they are cultured spatially separated but in close proximity. At day 35 of hOB culture and mineralization on cryogel discs, starPEG–heparin hydrogel monoculture models (PEG–MMP γ = 1; PEG–MMP–GFOGER γ = 1.25) or triculture breast-cancer models were prepared as described above. Immediately after preparation, the hydrogel droplets were attached to the cryogels. Therefore, 6 µL of the same hydrogel material with the same cross-linking degree (without cells) was prepared as a ‘hydrogel glue’ and instantly applied to the surface of the swollen cryogel. Afterwards, the flat side of the cell-laden hydrogel was put on the top and the construct was secure within 2 min. The 3D–3D coculture constructs were cultured in medium of the corresponding hydrogel cell type (monocultures: breast-cancer medium, tricultures: ECGM) and incubated at 37 °C and 5% CO_2_. Tricultures were photographed and fixed for immunofluorescence staining after 7 d of coculture. Monocultures were fixed after 21 d. 

## 5. Conclusions

3D breast-cancer models provide invaluable tools for the study of cell–cell and cell–matrix interactions. It was demonstrated here that starPEG–heparin hydrogels and cryogels provide highly useful tools for studying breast cancer and metastasis to bone. The models presented here offer a high degree of flexibility of design and stability with up to four cell types for application in various bone metastatic malignancies. Several characteristics of the cells (morphological changes, proliferation, migration, stability) in the different models were explored in the current study, and proof of the practicability of these models was demonstrated. In the future, the 3D–3D co-construct could be adopted for culturing other cell types, e.g., osteoclasts, in the direct coculture construction or other cancer cells which are prone to bone metastases. The established biphasic 3D models offer promise for the study of the molecular mechanisms of bone metastasis, and may also be extended to study patient-derived breast-cancer cells for personalized medicine and evaluate individual metastatic potential. 

## Supplementary Materials

The following are available online at http://www.mdpi.com/2072-6694/10/9/292/s1. Supplementary Figure S1, viability and mean spheroid diameter of cells growing in hydrogels with varying molar ratio. Supplementary Figure S2, immunofluorescent staining of MCF-7 and MDA-MB-231 cells in PEG–MMP (no ligand) and PEG–MMP–GFOGER hydrogels. Supplementary Figure S3, Alizarin red staining of hOBs grown in 2D. Supplementary Figure S4, appearance and properties of the starPEG–heparin cryogel scaffolds used in this study. Supplementary Figure S5, hOB cultures in cryogel construct. Figure S6: light microscopy images of MDA-MB-231 cultured on hOBs in a 2D–3D coculture model. Supplementary Figure S7, microscopic pictures of MCF-7 and MDA-MB-231 tricultures with HUVECs and MSCs grown in hydrogels on hOBs in a 2D–3D coculture model. Supplementary Figure S8, MCF-7 and MDA-MB-231 tricultures grown in a 3D–3D coculture model.
