# Supplementary material for: Three-Dimensional In Vitro Hydro- and Cryogel-Based Cell-Culture Models for the Study of Breast-Cancer Metastasis to Bone
Source: Cancers (Basel). 2018 Aug 27;10(9):292. doi: 10.3390/cancers10090292 (PMC6162532; doi:10.3390/cancers10090292)
Supplement: Supplementary file 1 [file cancers-10-00292-s001.pdf]

Three-Dimensional In Vitro Hydro- and Cryogel-Based Cell-Culture Models for the Study of Breast-Cancer Metastasis to Bone

Laura J. Bray, Constanze Secker, Berline Murekatete, Jana Sievers, Marcus Binner, Petra B. Welzel, and Carsten Werner

Supplementary Material

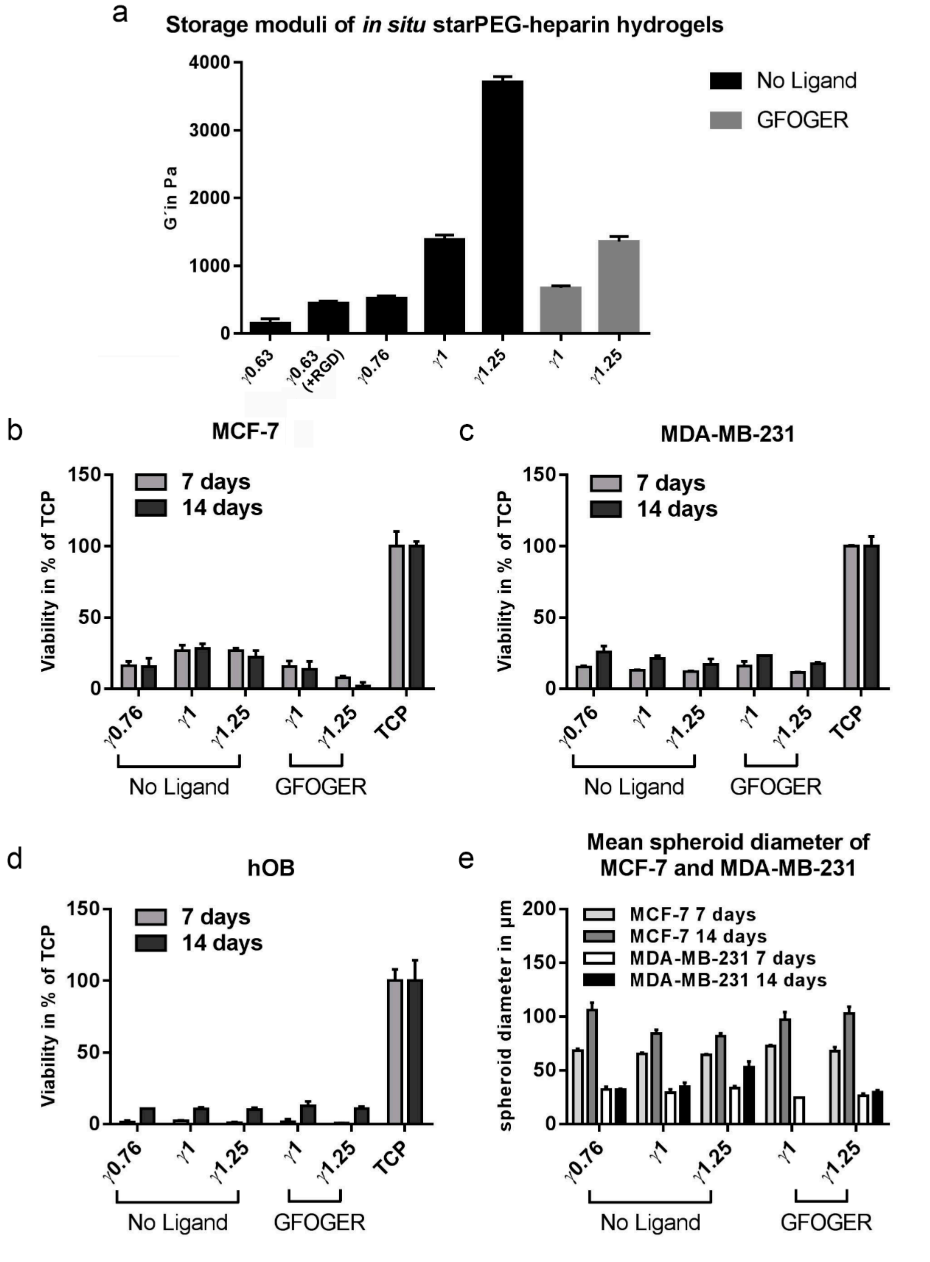

Figure S1. Viability and mean spheroid diameter of cells growing in hydrogels with varying molar ratio (γ) of starPEG: heparin. (Storage moduli of in hydrogels (2 technical replicates per condition, n = 1, measured at different strains (2–10 %), data shown as mean ± SD). (b–d) Viability was assessed with PrestoBlue assays after 7 and 14 d of culture (3 technical replicates per condition, assay performed in duplicate, mean ± SEM, n = 1). (e) Spheroid diameter of tumor cell lines are presented as mean ± SEM (3 technical replicates per condition, 3 photographs taken per technical replicate, n = 1).

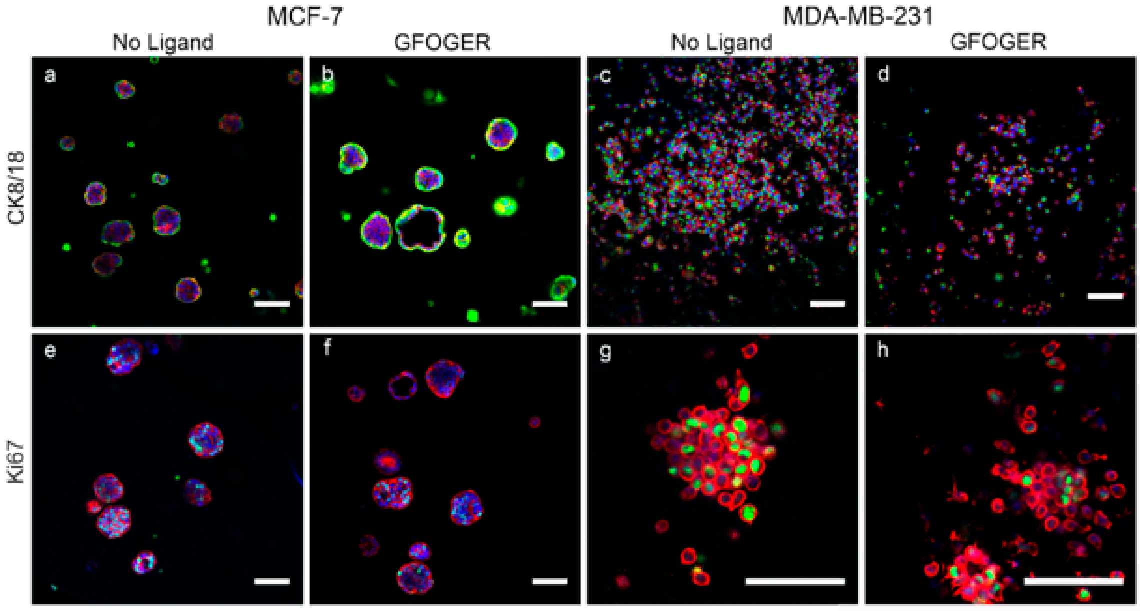

Figure S2. Immunofluorescent staining of MCF-7 and MDA-MB-231 cells in PEG-MMP (no ligand) and PEG-MMP-GFOGER hydrogels. (a–d) Expression of cytokeratin 8/18 (green; epithelial marker) on (a, b) MCF-7 and (c, d) MDA-MB-231 cells after 14 d 3D culture. e-h) Expression of Ki67 (swapped) (green; proliferative marker) on (e, f) MCF-7 and (g, h) MDA-MB-231 cells after 14 d 3D culture. Counter staining was performed for actin (red) and nuclei (blue). Scale bar = 100 µm.

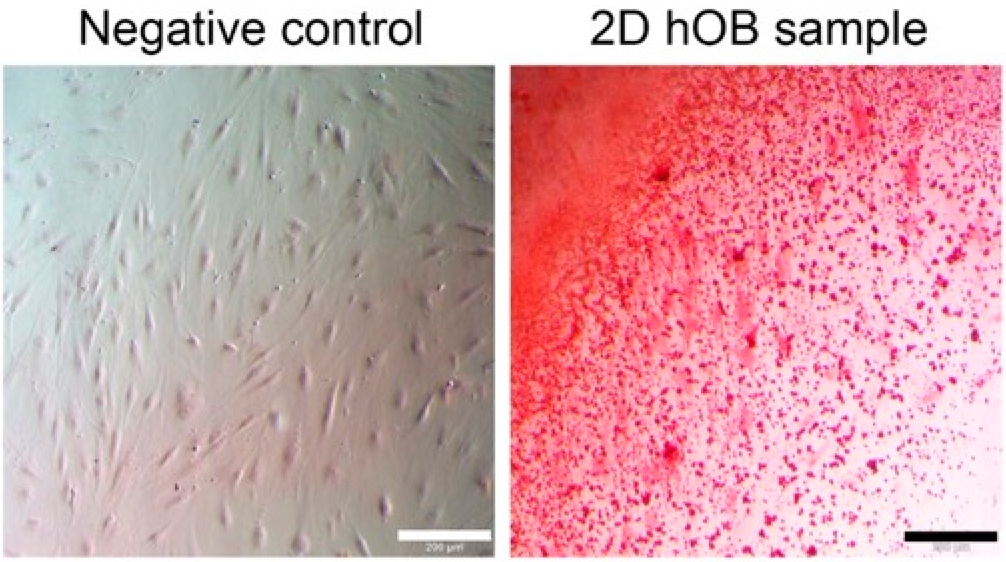

Figure S3. Alizarin Red staining of hOBs grown in 2D. Left image shows hOBs grown for 7 d in proliferation medium as a negative control. Right image shows hOBs cultivated for 7 d in proliferation medium and then mineralization medium was administered for 10 d. The bright red calcium deposits indicate osteoblastic mineralized matrix formation. Scale bar = 200 µm.

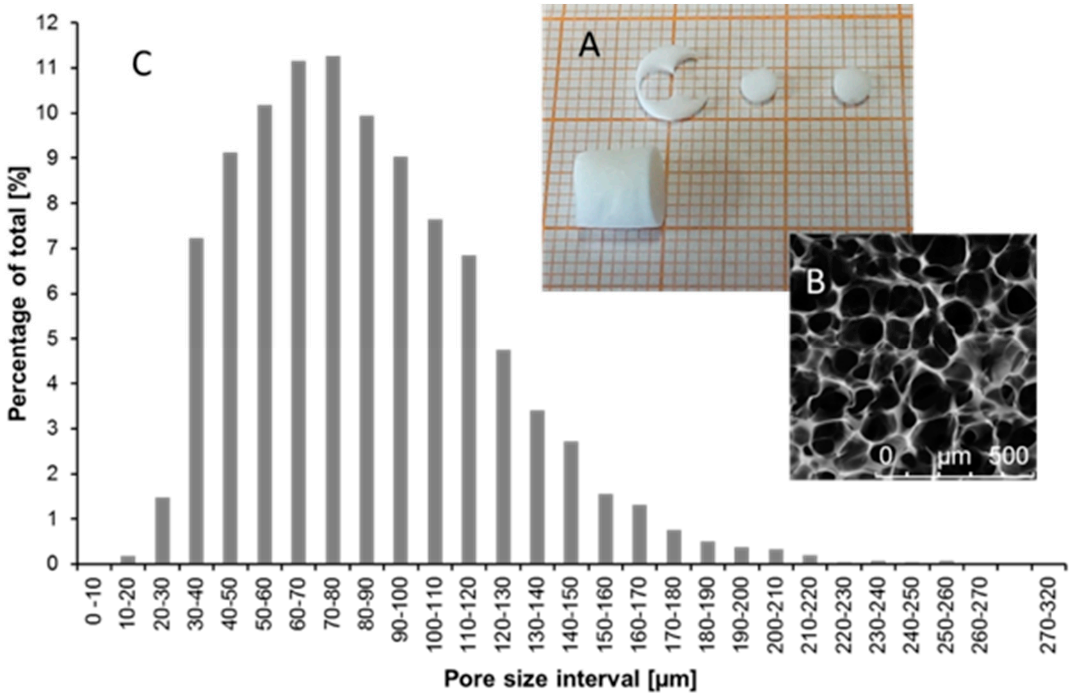

Figure S4. Appearance and properties of the starPEG-heparin cryogel scaffolds used in this study. (**A**) Representative digital image of a dry cryogel cylinder, a cut disc and two punched scaffolds. (**B**) Representative scanning confocal image of a fluorescence labeled PBS swollen cryogel section (light-colored). Scale bar = 500 µm. (**C**) Pore size distribution of the PBS swollen cryogel scaffolds (5235 pore dimensions were measured from n = 9 scaffolds).

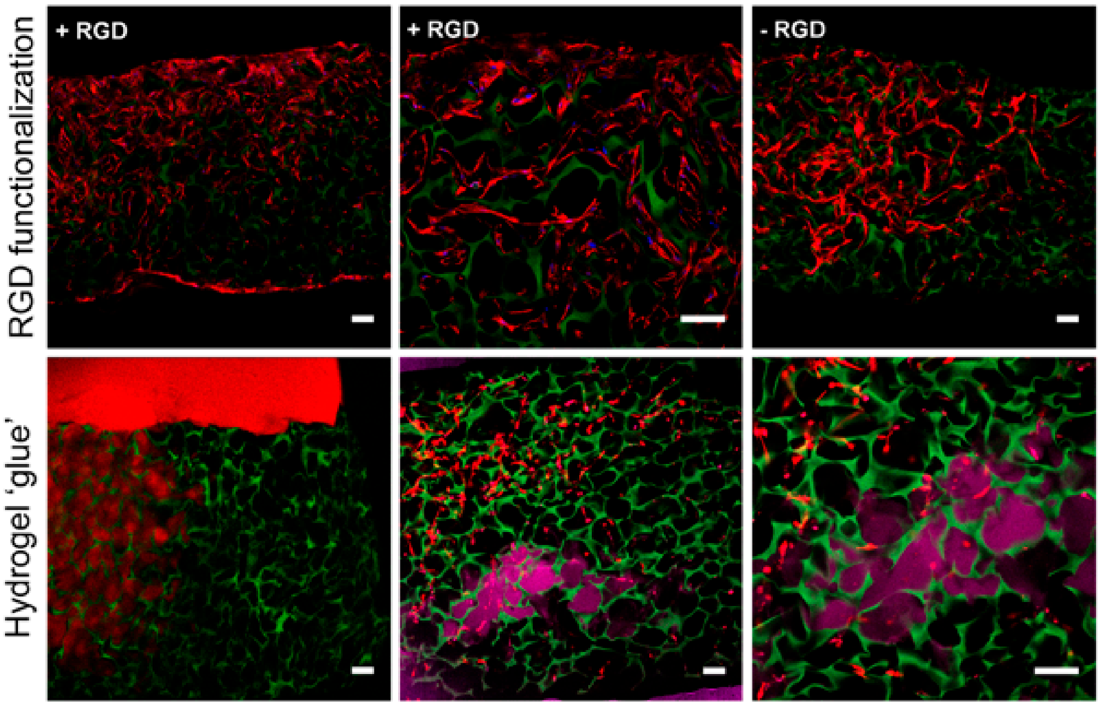

Figure S5. HOB cultures in cryogel construct. Alexa Fluor 488 (green) labeled cryogels were used for visability. Pictures in the upper row depict the hOBs (cell staining: actin (red), nuclei (blue)) adhesive behavior in the RGD functionalized or non-functionalized cryogels after 14 d. The lower row shows images with in situ starPEG heparin hydrogel glue. Lower left: the hydrogel glue (Alexa Fluor 647 labeled, red) was deeply absorbed into the macropores in the middle of the spongy cryogel (without cells). Both lower right pictures show hOBs within the cryogel and the hydrogel glue (Alexa Fluor 555 labeled, purple) which is partly dissolved after 14 d. Scale bar = 100 µm.

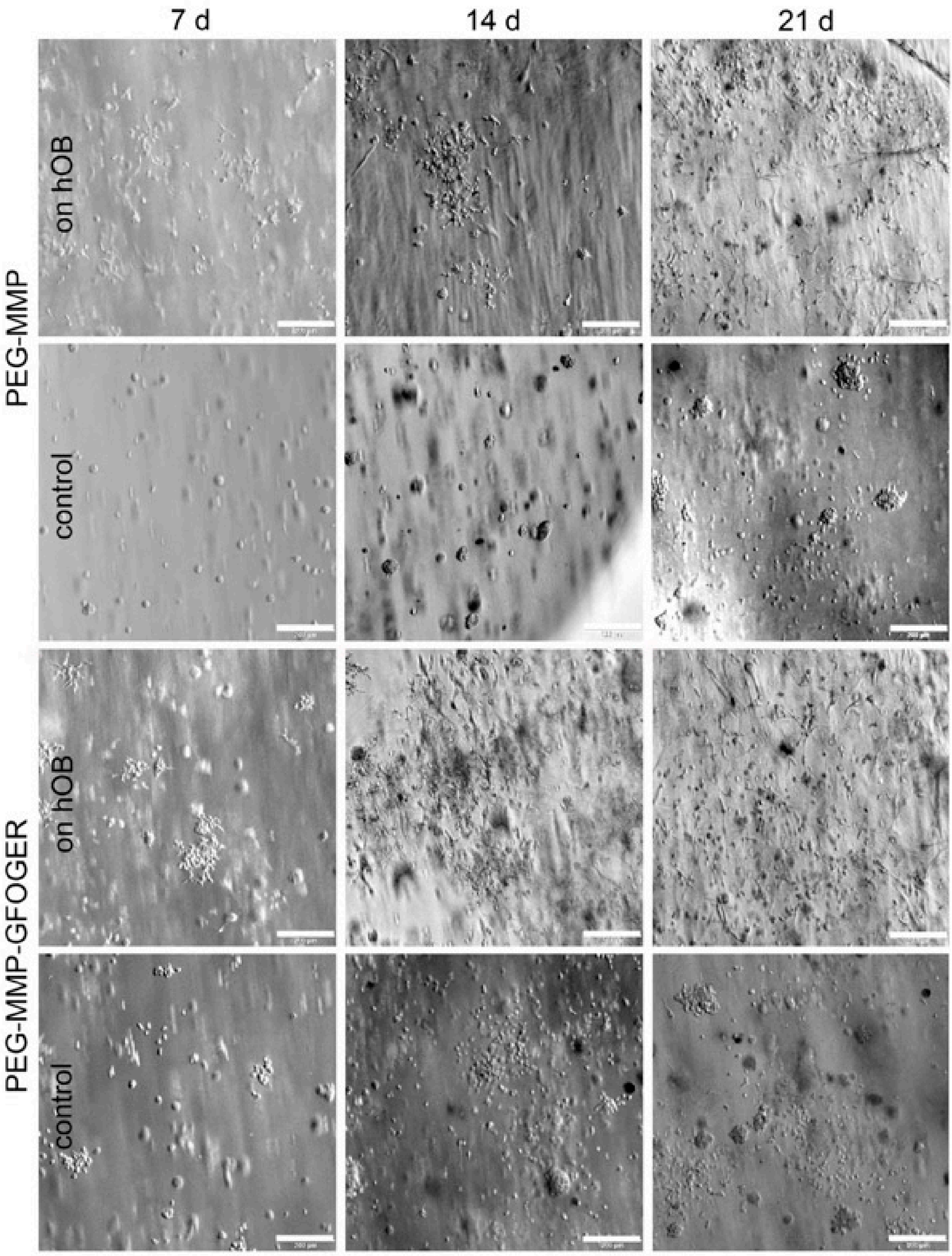

Figure S6. Light microscopy images of MDA-MB-231 cultured on hOBs in a 2D–3D co-culture model. Cells were cultured for 7 d, 14 d and 21 d in either PEG-MMP (γ = 1) or PEG-MMP-GFOGER (γ = 1.25). Scale bar = 200 μm.

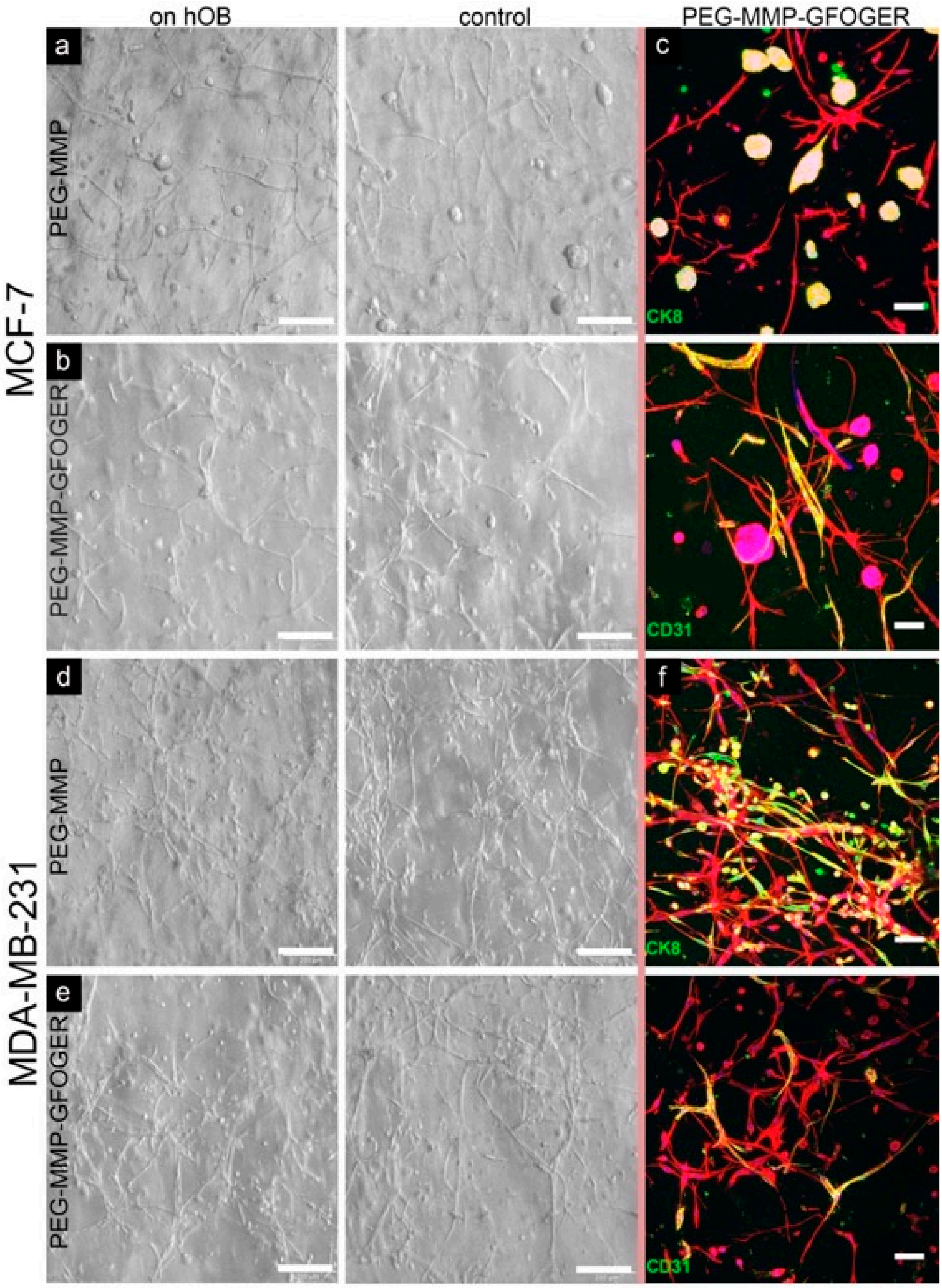

Figure S7. Microscopic pictures of MCF-7 (a–c) and MDA-MB-231 (d–f) tri-cultures with HUVECs and MSCs grown in hydrogels on hOBs in a 2D–3D co-culture model. Cells were either cultured in PEG-MMP or PEG-MMP-GFOGER hydrogels for 7 d. Brightfield images show network formation in both examined groups (on hOBs or without hOBs) as well as in both hydrogel types (scale bar = 200 µm). The 3D confocal image projections show actin (red), nuclei (blue) and immunofluorescence staining of CK8 and CD31 (green) of PEG-MMP-GFOGER control gels as examples. Scale bar = 100 µm.

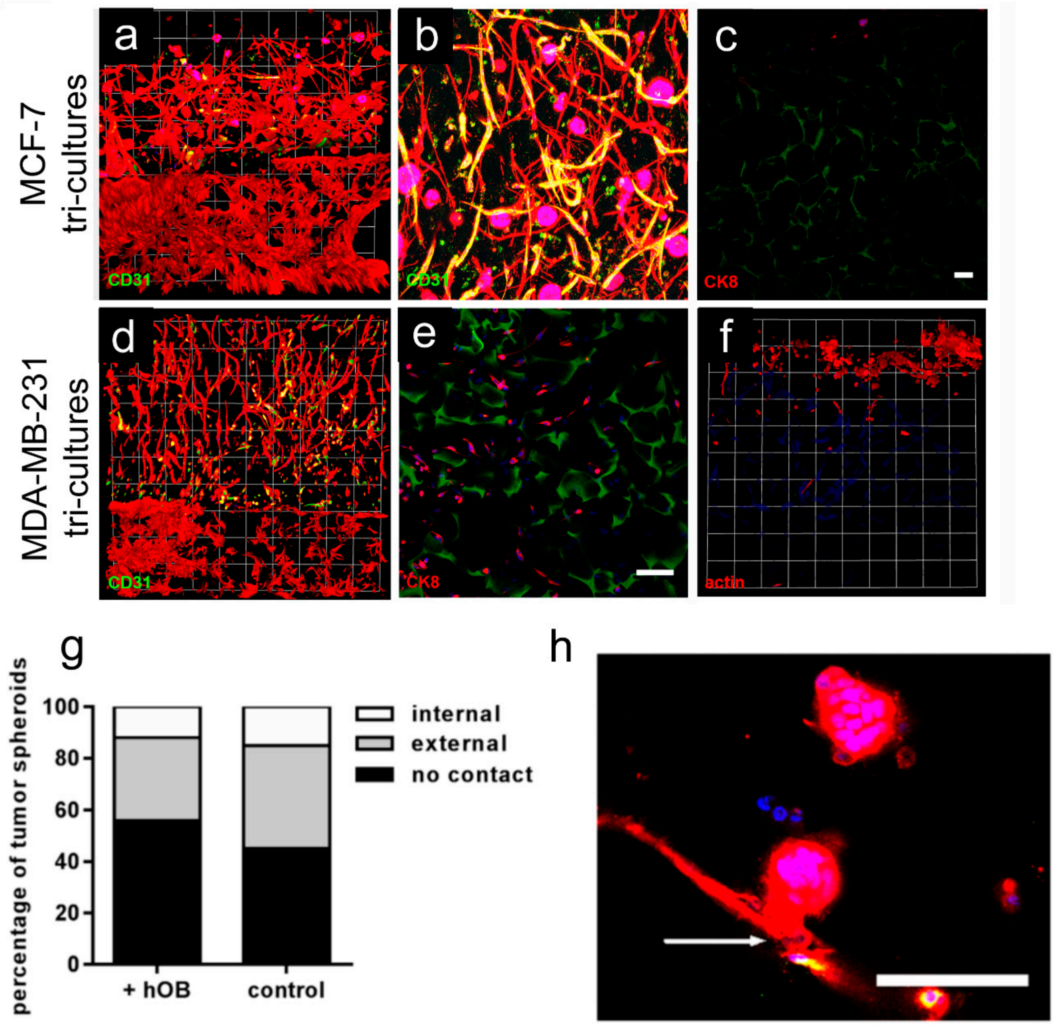

Figure S8. MCF-7 and MDA-MB-231 tri-cultures grown in a 3D–3D co-culture model. (g) Contact points of MCF-7 and HUVECs were classified as (1) internal contact, (2) external contact and (3) no contact. The numbers of tumor spheroids are plotted as percentages of each category (cryogel with hOB: n=3, 59 spheroids, empty control cryogel (n = 2, 40 spheroids). (h) Confocal microscopy picture reveals a representative example of an external contact (indicated by the white arrow) and a tumor without vascular contact. Colors represent actin (red), nuclei (blue) and immunofluorescence staining of CD31 (green). Scale bar = 100 µm.
